# Complementary Post Transcriptional Regulatory Information is Detected by PUNCH-P and Ribosome Profiling

**DOI:** 10.1038/srep21635

**Published:** 2016-02-22

**Authors:** Hadas Zur, Ranen Aviner, Tamir Tuller

**Affiliations:** 1 Department of Biomedical Engineering, the Engineering Faculty, Tel Aviv University, Israel; 2 Department of Cell Research and Immunology, Tel Aviv University, Israel; 3 The Sagol School of Neuroscience, Tel Aviv University, Israel

## Abstract

Two novel approaches were recently suggested for genome-wide identification of protein aspects synthesized at a given time. Ribo-Seq is based on sequencing all the ribosome protected mRNA fragments in a cell, while PUNCH-P is based on mass-spectrometric analysis of only newly synthesized proteins. Here we describe the first Ribo-Seq/PUNCH-P comparison via the analysis of mammalian cells during the cell-cycle for detecting relevant differentially expressed genes between G1 and M phase. Our analyses suggest that the two approaches significantly overlap with each other. However, we demonstrate that there are biologically meaningful proteins/genes that can be detected to be post-transcriptionally regulated during the mammalian cell cycle only by each of the approaches, or their consolidation. Such gene sets are enriched with proteins known to be related to intra-cellular signalling pathways such as central cell cycle processes, central gene expression regulation processes, processes related to chromosome segregation, DNA damage, and replication, that are post-transcriptionally regulated during the mammalian cell cycle. Moreover, we show that combining the approaches better predicts steady state changes in protein abundance. The results reported here support the conjecture that for gaining a full post-transcriptional regulation picture one should integrate the two approaches.

Gene expression is a multi-step process, with the first stage of this process (transcription) and its product (mRNA levels) comprehensively studied and measured. However, it was shown that the correlation between mRNA and protein levels is relatively limited^1^,^2^,^3^. Consequently, recently various technologies for studying post transcriptional regulation, and specifically translation, have emerged to close this gap^1^,^3^,^4^,^5^,^6^,^7^,^8^,^9^,^10^,^11^,^12^,^13^,^14^,^15^,^16^,^17^,^18^,^19^,^20^,^21^,^22^,^23^,^24^,^25^,^26^.

Currently the most common technology for studying translation is ribosomal profiling (Ribo-Seq). Although ribosomal profiling was introduced only several years ago it has already been successfully employed for answering fundamental biological questions related to post transcriptional regulation of gene expression^5^,^27^,^28^,^29^,^30^. Figure 1A includes the major steps of the ribosomal profiling approach: Cells are treated with cycloheximide (or a different drug) to arrest translating ribosomes; extracts from these cells are then treated with RNase to degrade regions of mRNAs not protected by ribosomes; the resulting 80S monosomes, many of which contain a ~30-nucleotide ribosomal protected footprint (RPF), are purified (e.g. using sucrose cushion) and then treated to release the RPFs, which are processed for Illumina high-throughput sequencing. The next steps are computational: the RPFs are mapped to the transcriptome, and based on them it is possible to infer various biophysical properties related to the translation elongation process. For example, each ribosomal footprint read is related to a certain codon along the mRNA, and was generated when the codon in one of the mRNA molecules is covered by a ribosome. Thus, from a biophysical perspective, relatively slower codons along the mRNA can be detected based on the fact that they are covered by ribosomes for longer periods of time, creating a higher number of reads.

Recently a new approach called PUNCH-P^31^,^32^ was proposed. This approach is based on the combination of biotinylated puromycin with MS analysis to globally label *newly* synthesized proteins, enabling identifying the proteins translated in a certain condition. The method involves isolation of ribosomes by ultracentrifugation followed by cell-free labeling of nascent polypeptide chains with 5′ biotin-dC-puromycin 3′ (Biot-PU), capture on immobilized streptavidin, and analysis by liquid chromatography-tandem MS (LC-MS/MS). This work flow leads to the identification of thousands of newly synthesized proteins in a certain condition, generating a snapshot of the cellular translatome, see Fig. 1B.

It is easy to see that both approaches measure very similar but *non-identical* aspects related to protein synthesis (Fig. 1A,B). Roughly, Ribo-Seq is based on the total number of ribosomes on the mRNA molecules related to a certain gene; PUNCH-P, on the other hand, is based on the total amount of *nascent peptide* emerging from the ribosomes on the mRNA molecules related to a certain gene which are *translating* at the time of the experiment. Since not all ribosomes on the mRNA (i.e. can be detected by Ribo-Seq) are actually translating^33^,^34^,^35^ at a certain moment (i.e. can be detected by PUNCH-P), the signal detected by these approaches is not identical.

Furthermore, the different approaches are expected to have different experimental biases/noise as they are based on different experimental/analysis techniques: sequencing vs. proteomics.

It is important to mention that a priori it is not clear which approach (or if there is an approach that) performs better. This is true not only due to the different biases related to the different methods, but also due to the fact that each of them is expected to capture biological meaningful signals not detected by the other: changes in the number of translating ribosomes, but also the total number of ribosomes, are expected to be relevant to protein levels regulation.

Thus, the aim of this study is to compare these two methods, which were both performed at the G1 and M phases of the cell cycle, and discern if their integration can yield improved predictions of relevant genes/proteins, and uncover otherwise elusive biological phenomena. To this end we: 1. Tested the predictive power of steady-state protein levels of each approach and their combination (Fig. 1C). 2. Uncovered significant M/G1 differentially expressed genes with each of the approaches. 3. Exploiting these genes we discovered relevant intra-cellular pathways with each of the approaches (Fig. 1E). 4. Discerned biological relevant properties related to the differentially expressed genes detected by each approach and the protein-protein interaction network (Fig. 1D,F). In points 2.-3. we specifically studied the genes/proteins detected by *only one* of the methods.

Data for PUNCH-P was taken from^31^ (see Methods), while we generated the Ribo-Seq data via two experiments, one with 3 replicates, and the other with one replicate, totalling 4 technical replicates per each cell-cycle phase, G1 and M (see Methods and Supplementary Methods).

## Results

### Correlations based on Ribo-Seq and PUNCH-P with steady state protein levels

Steady state protein levels are expected to be affected by all gene expression steps (e.g. transcription, translation, mRNA degradation, protein degradation). Thus, steady state protein levels (PSS) are expected to correlate with mRNA levels, PUNCH-P (PP), and Ribo-Seq (RP). In addition, it is easy to see that RP and PP (which encapsulate both the mRNA levels and the translation step, or the number of ribosomes on the mRNA molecules) are expected to have higher correlation than mRNA levels with steady state protein levels. Moreover we expect to see relatively high correlation between PP and RP as they measure similar variables. Finally, we also expect that the combination of the different measures can improve the prediction of steady state proteins, as each of them encapsulates non-identical aspects of gene expression and exhibits different experimental biases. All these points are verified in this sub-section.

**Figure 2:**
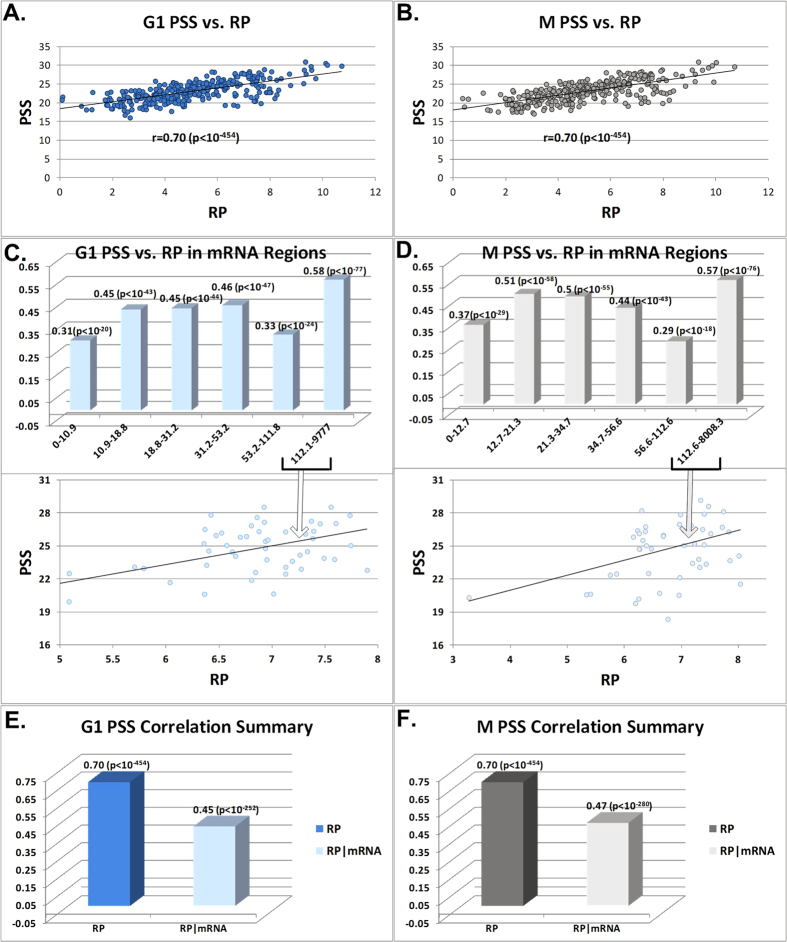
(**A**) Scatter plot of steady state protein levels (PSS) (y-axis , data is log2-scaled) and Ribo-Seq (RP) (x-axis, read count log2-scaled RPKM (see Methods)) G1 phase. (**B**) Scatter plot of PSS (y-axis log2(intensity)) and RP levels (x-axis, read count log2-scaled RPKM (see Methods)) M phase. (**C**) Correlation between PSS and RP G1 phase for different bins of genes sorted by mRNA levels (the y-axis is the correlation, the x-axis is the mRNA levels RPKM ranges, scatter plots are log2-scaled). (**D)** Correlation between PSS and RP M phase for different bins of genes sorted by mRNA levels (the y-axis is the correlation, the x-axis is the mRNA levels RPKM ranges, scatter plots are log2-scaled). (**E**) A summary of the 2 correlations performed with PSS: RP, and RP controlled for mRNA levels (partial correlation) for G1 phase. F. A summary of the 2 correlations performed with PSS: RP, and RP controlled for mRNA levels (partial correlation) for M phase.

**Figure 3:**
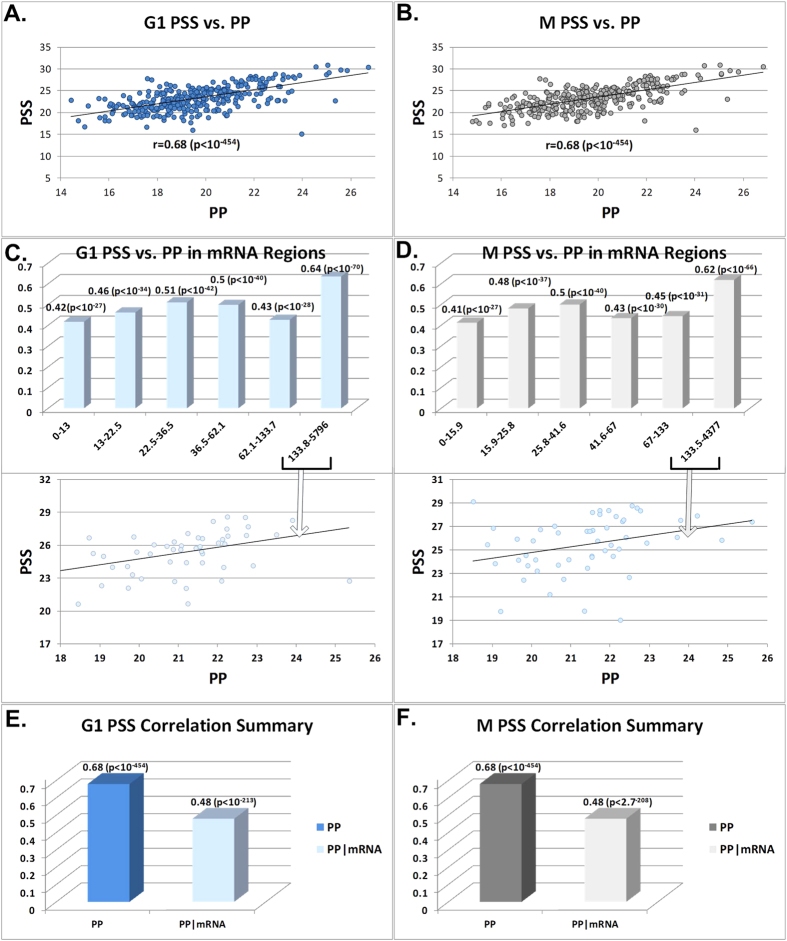
(**A**) Scatter plot of steady state protein levels (PSS) (y-axis log2(intensity)) and PUNCH-P (PP) (x-axis log2(intensity)) G1 phase. (**B**) Scatter plot of PSS (y-axis log2(intensity)) and PP levels (y-axis log2(intensity)) M phase. (**C**) Correlation between PSS and PP G1 phase for different bins of genes sorted by mRNA levels (the y-axis is the correlation, the x-axis is the mRNA levels RPKM ranges, scatter plots are log2-scaled). (**D**) Correlation between PSS and PP M phase for different bins of genes sorted by mRNA levels (the y-axis is the correlation, the x-axis is the mRNA levels RPKM ranges, scatter plots are log2-scaled). (**E**) A summary of the 2 correlations performed with PSS: PP, and PP controlled for mRNA levels (partial correlation) for G1 phase. (**E**) A summary of the 2 correlations performed with PSS: PP, and PP controlled for mRNA levels (partial correlation) for M phase. PP data is log scaled.

At the first step, we aimed at providing estimations for the effect of transcription and translation on steady state protein levels via the correlation of the products of these stages. Our analyses demonstrate that the correlation (all correlations reported in the paper are Spearman, see Methods) between the G1 and M phases of steady state protein levels and Ribo-Seq (r(PSS,RP)) are: 0.70 (p < 10^−454^) (see Fig. 2A,E) and 0.70 (p < 10^−454^) respectively (see Fig. 2B,F); the correlation is significant and high also when controlling for mRNA levels (r (PSS,RP|mRNA): 0.45 (p = 2.4·10^−252^) (see Fig. 2C,E) and 0.47 (p = 4·10^−280^) (see Fig. 2D,F). The correlation between M and G1 phases of steady state protein levels and PUNCH-P (r(PSS,PP)) are: 0.68 (p < 10^−454^) (see Fig. 3A,E) and 0.68 (p < 10^−454^) (see Fig. 3B,F); the correlation is significant and high also when controlling for mRNA levels (r (PSS,PP|mRNA): 0.48 (p = 3.3·10^−213^) (see Fig. 3C,E) and 0.48 (p = 2.7·10^−208^) (see Fig. 3D,F). The correlation of PSS with mRNA levels is indeed lower than with both RP and PP, (r(PSS,mRNA)): 0.61 (p < 10^−454^) and 0.60 (p < 10^−454^), for the G1 and M phases respectively (see Supplementary Figure S4).

**Figure 4:**
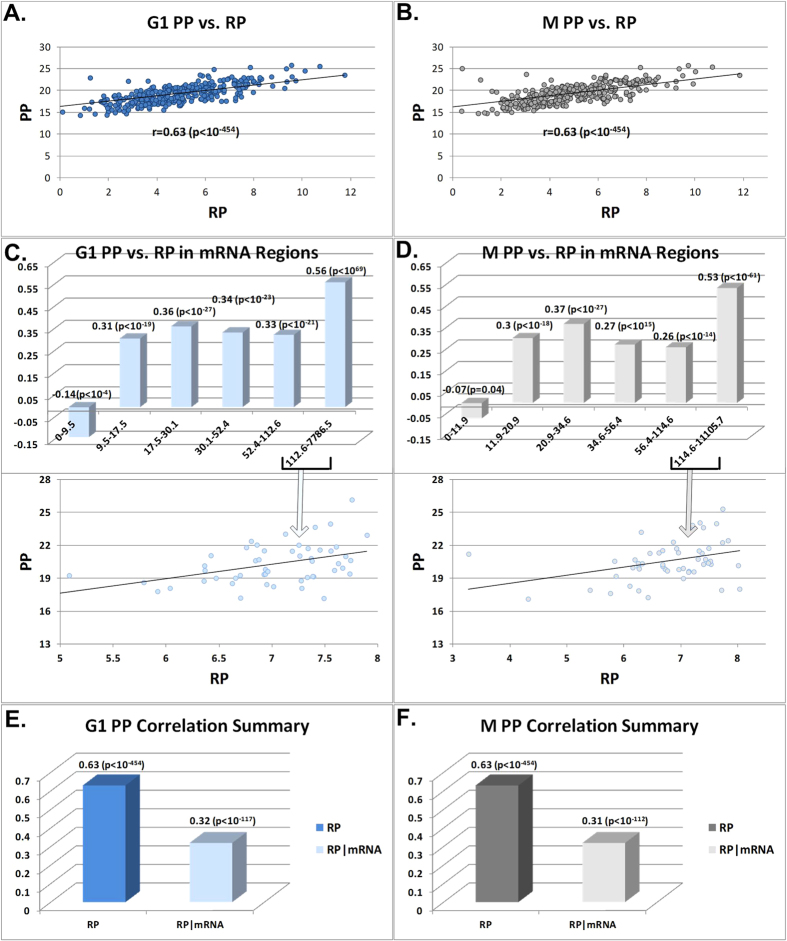
(**A**) Scatter plot of PUNCH-P (PP) (y-axis log2(intensity)) and Ribo-Seq (RP) G1 phase (x-axis, read count log2-scaled RPKM (see Methods)). (**B**) Scatter plot of PP (y-axis log2(intensity)) and RP levels M phase (x-axis, read count log2-scaled RPKM (see Methods)). (**C**) Correlation between PP and RP G1 phase for different bins of genes sorted by mRNA levels (the y-axis is the correlation, the x-axis is the mRNA levels RPKM ranges, scatter plots are log2-scaled). (**D**) Correlation between PP and RP M phase for different bins of genes sorted by mRNA levels (the y-axis is the correlation, the x-axis is the mRNA levels RPKM ranges, scatter plots are log2-scaled). (**E**) A summary of the 2 correlations performed with PP: RP, and RP controlled for mRNA levels (partial correlation) for G1 phase. (**E**) A summary of the 2 correlations performed with PP: RP, and RP controlled for mRNA levels (partial correlation) for M phase. PP data is log scaled.

Next we estimated the correlation between the two methods PP and RP to evaluate the similarity between the prediction obtained by the two methods. Our analyses demonstrate that the correlations between the M and G1 phases of PUNCH-P and Ribo-Seq are 0.63 (p < 10^−454^) (see Fig. 4A,E) and 0.63 (p < 10^−454^) (see Fig. 4B,F) respectively; the correlations are high also when controlling for mRNA levels (r (PP,RP|mRNA)): 0.31 (p = 2.5·10^−112^) (see Fig. 4C,E) and 0.32 (p = 2.2·10^−117^) (see Fig. 4D,F) for M and G1 respectively.

**Figure 5:**
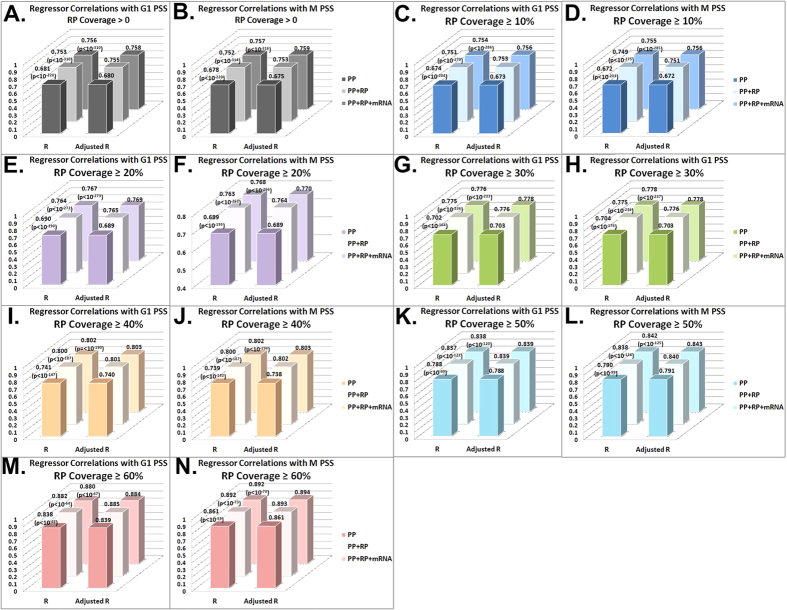
Correlations with M and G1 steady state protein levels (PSS) for three regressors based on PP, PP and RP, and PP, RP and mRNA respectively, as a function of the RP coverage (>0 – 60%), the y-axis is the correlation. We performed a 2-fold cross validation 100 times per Spearman linear regressor, with the standard deviation of all the regressors being between 0.0062–0.0141, with the variation being lower for the most part as coverage increases and with more measurements combined. One can see that combining all 3 measurements improves correlations with steady state protein levels. See Supplementary file Supplementary_Table_S1_RegressorCorrs.xlsx.

Finally, as can be seen in Fig. 5, regressors based on PP and RP for the M and G1 phases (see Methods), as a function of RP coverage from >0 to ≥60%, achieve improved correlation with steady state protein levels in comparison to a regressor based only on either PP or RP, while including mRNA levels further improves the correlation (but not substantially), see Supplementary file Supplementary_Table_S1_RegressorCorrs.xlsx.

The results demonstrate that the correlation between PP and RP is high (as expected) but is far from being perfect. In addition, these results support the hypothesis that the variance in protein levels can be explained by PP, RP, and mRNA levels; thus both changes in mRNA levels (regulated among others via transcription) and changes in ribosomal densities (as part of the translation step) effect the changes in protein abundance (translation, and not only transcription, as traditionally thought, has important contribution to changes in protein levels). The results also show that PP and RP have significant predictive power of protein levels. Finally, we demonstrate how a regression based both on PP and RP improves the prediction of steady state protein levels. Since steady state protein levels may also be affected by proteins not translated at the moment of the experiment, a predictor based on both PP and RP improves the prediction of steady state protein levels upon a predictor based on PP or RP alone.

### There are relevant genes detected to be differentially expressed exclusively by each method

At the next step our objective was to show that both PP and RP can be used for detecting relevant differentially transcriptional and post transcriptional regulated genes, and that each of these methods exclusively detects relevant genes.

To demonstrate this point we first inferred the set of differentially expressed (DE) genes between the G1 and M phases of the cell cycle detected for PUNCH-P (PP) and Ribo-Seq (RP) separately. M/G1 differentially expressed (DE) genes were determined according to DESeq^36^ for Ribo-Seq (RP), where the top 10% most significant FDR p-values were selected (See Methods and Supplementary Methods), and for PUNCH-P (PP) according the top 10% ANOVA significant fold change (see Methods,^31^). At the next step we defined three DE gene groups: 1. RP-PP (genes that are significantly DE in RP but not in PP; 1,090 genes). 2. PP-RP (genes that are significantly DE in PP but not in RP; 200 genes). 3. RP∩PP (genes that are significantly DE both in PP and in RP; 125 genes). These two DE sets, and the three DE groups derived from them will be employed throughout the paper. We performed pathway and biological process enrichment for each of the groups (Methods). To achieve our objective, we aimed to show that relevant pathways and biological processes are significantly enriched with DE genes in all three cases.

**Figure 6:**
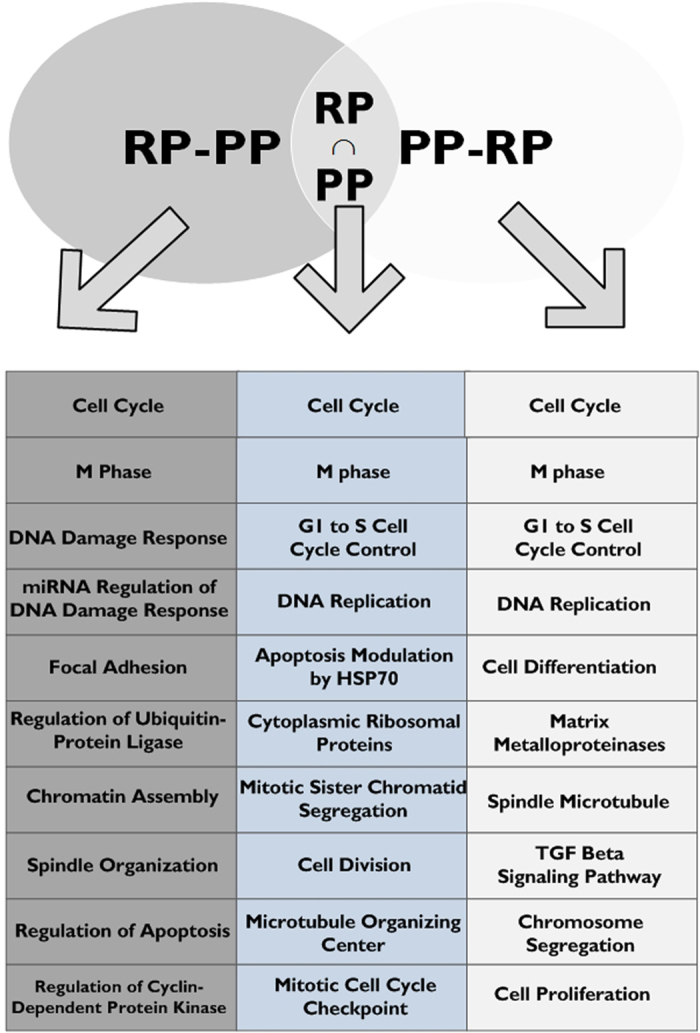
Selected pathways and biological processes which are significantly enriched by the 3 groups of DE genes (for a full pathway list please see Supplementary Information Table 1 (section 3.1), and for a full biological process list see Supplementary files Supplementary_Table_S3_RPDavidReports.xlsx, Supplementary_Table_S4_PPDavidReports.xlsx, Supplementary_Table_S5_RPiPPDavidReports.xlsx).

As can be seen in Fig. 6 (for a full pathway list please see Supplementary Information Table 1 (section 3.2), and for a full biological process list see Supplementary files Supplementary_Table_S3_RPDavidReports.xlsx, Supplementary_Table_S4_PPDavidReports.xlsx, Supplementary_Table_S5_RPiPPDavidReports.xlsx, see further details in Methods), each technique enables detecting meaningful genes/proteins that are not detectable by the other. The detected differentially expressed post-translational regulatory pathways related to the three sets described via enrichment analysis include: central cell cycle, central gene expression regulation, DNA damage and replication, and chromosome arrangement.

For example, all three sets are enriched with genes related to the cell cycle and M phase; RP-PP and RP∩PP are enriched with genes related to apoptosis regulation, while PP-RP is enriched with genes related to cell proliferation; RP-PP is enriched with genes related to Spindle Organization and DNA Damage response, while PP-RP and RP∩PP are enriched with genes related to Spindle Microtubule/Microtubule organization center and DNA replication.

We would like to emphasize the fact that aside from detecting distinct biologically relevant pathway enrichments, there are cases that the sets RP-PP and PP-RP are enriched with genes related to the same (or very similar) pathways, suggesting that the different techniques tend to find different parts of the same relevant pathways. This evidence again demonstrates the advantage of combining/considering the two methods.

Now, in order to further demonstrate that each of the techniques, RP and PP, uncovers biologically relevant protein-protein interactions that cannot be detected by the other technique, three PPI network colouring schemes were defined, where “black” nodes represent differentially expressed genes (DE; see Methods) as above between the G1 and M phases of the cell cycle. In the first case, the black nodes were defined as genes that are DE according to RP but not PP (RP-PP); in the second case the black nodes were defined as genes that are DE according to PP but not RP (PP-RP); in the third case the black nodes were defined as genes that are DE according to both RP and PP; similarly to the previous analysis.

**Figure 7:**
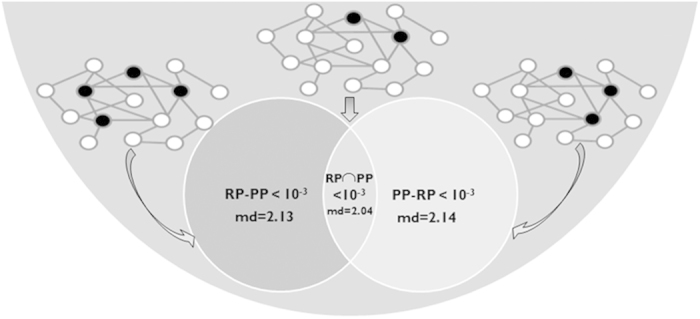
Three PPI network colouring schemes were defined, where black nodes represent DE genes (based on PP and/or RP): 1. RP-PP. 2. PP-RP. 3. RP∩PP DE. We compute the mean distance (md) in each between all black nodes. For each case we compute a PPI empirical p-value by randomizing each PPI network 100 times respectively and calculating the black node distance. Shorter distances between DE PPI nodes means more meaningful biological signals (we expect to see physical interactions between DE genes).

We computed the mean distance (md) between all black nodes in each of the aforementioned three cases. For each case, we computed a PPI empirical p-value by randomizing each PPI network 100 times respectively generating random networks with a similar degree distribution as the original one, and calculating the black node distance, showing that the mean distances are shorter in the real graph in comparison to the random ones (see details in the Methods section). Shorter distances between DE PPI nodes means more meaningful biological signals, as if indeed we uncover real regulatory changes in signalling pathways, we expect them to be clustered/close in the PPI network (we expect to see physical interactions between DE genes). All p-values were <10^−2^ (when 100 permutations are performed a p-value <10^−2^ means that the observed distance was always shorter than the distances obtained during all 100 random permutations), with the mean distance being shorter (2.01) in the case of the RP∩PP than in the case of the RP-PP and the PP-RP groups (2.12 and 2.13, respectively) (see Fig. 7).

Our analyses demonstrate that genes detected by each of the methods (even if not detected by the other) tend to be closer to each other than expected by the null model in the PPI network. Thus, this result supports the hypothesis that not all biological meaningful genes detected by one of the methods are detected by the other.

### Modules of differentially post-transcriptionally expressed genes and physical interactions

**Figure 8:**
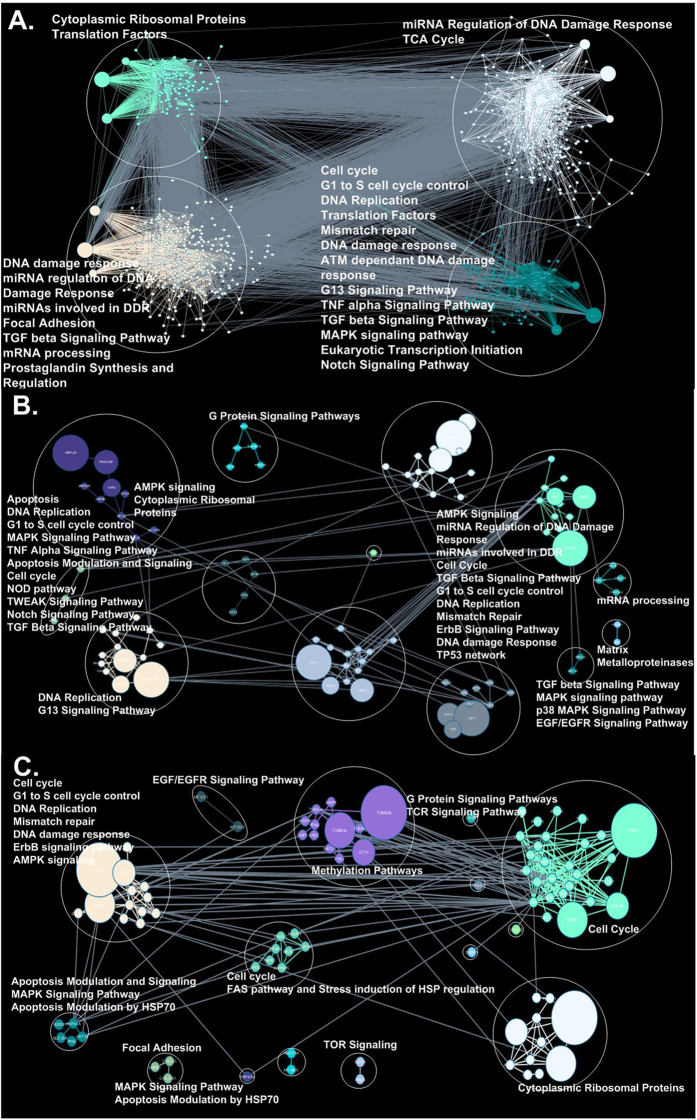
(**A**) RP-PP clusters: 879 genes participate, resulting in 4 clusters. (**B**) PP-RP clusters: 96 genes participate, resulting in 13 clusters. (**C**) RP∩PP clusters: 90 genes participate, resulting in 15 clusters. The functional enrichment related to each cluster appears in the figure. There are 4 node sizes depicted in the figure, according to their centrality (the 4^th^ size being equal for most nodes is a coarse-grained portrayal for simplicity). For the full cluster pathway enrichment see Supplementary_Table_S6_ClusterPathwayEnrichment.xlsx.

To better understand the differentially expressed genes detected by PP and RP we performed a clustering analysis (Newman algorithm^37^, see Methods), on the PPI network using the previously described DE genes according to RP and PP respectively, divided into the following three aforementioned groups: 1. RP-PP. 2. PP-RP. 3. RP∩PP (See Supplementary Figure S5 for RP∪PP (Supplementary section 3.3)). We projected each of the 3 groups on to the PPI network respectively, and only selected genes from each group that have a neighbour in that group in the PPI. In each case, the Newman algorithm partitions the PPI networks to sub-networks, where each sub-network is modular and includes nodes related only to the corresponding group. To understand the pathways related to each module we performed pathway enrichment based on the genes in each module (for all significantly enriched pathways see Supplementary file Supplementary_Table_S6_ClusterPathwayEnrichment.xlsx). As can be seen in Fig. 8, the number of modules detected for each of the groups RP-PP/PP-RP/RP∩PP were 4/13/15 respectively. The modules in all cases were enriched with relevant pathways related to the cell-cycle, DNA Damage and replication, and gene expression regulation and signalling. This analysis demonstrates again that meaningful sub networks of physical interactions are detected by each of the methods separately and together.

### Genes detected to be of *opposite* regulatory direction based on the different methods

Finally, we aimed to examine if there are genes that are detected to be significantly expressed based on both RP and PP but in *opposite* directions. To this end we looked at the following groups:

(a) Genes that have RP M/G1 fold-change >0 and PP M/G1 fold-change <0

(b) Gens that have RP M/G1 fold-change <0 and PP M/G1 fold-change >0

In total 78 genes appear in the first group and 68 genes in the second (the list of genes appears in Supplementary file Supplementary_Table_S7_RPopPPdiffGenes.xlsx). Both lists of genes were enriched with relevant pathways related to gene regulation and cell cycle (see Supplementary table 2 in Supplementary section 3.4). For example, the first group is enriched with genes related to DNA Replication and cell cycle control, while the second group is enriched with genes related to various central signalling pathways.

This result suggests that increasing/decreasing ribosomal density as detected by Ribo-Seq is not always related to increasing/decreasing the ribosomal density involved in protein synthesis at a certain time point as detected by Punch-P. There can be various explanations for this discrepancy which may be related (among others) to the fact that translation elongation (and not only translation initiation) is controlled during the mammalian cell cycle. For example, regulatory changes that cause ribosomal stalling during elongation^33^,^34^,^35^ may cause traffic jams, for example, near the beginning of the ORF where the ribosomes are not translating, or there is no nascent peptide emerging from the ribosome; since such ribosomes can theoretically be detected by RP and not PP they may increase RP but decrease PP. It is also possible that in some cases, due to traffic jams, the RNase does not accurately digest the mRNA between ribosome protected regions. This may result in underestimation of ribosome density and may lead to a decrease in *measured* ribosome density when the actual density increases (see, for example,^38^).

It is also important to emphasize that aspects related to changes in mRNA levels can’t trivially explain the observed discrepancies since both RP and PP are expected to be proportional to mRNA levels (if there are no traffic jams and biases).

### Details regarding some of the post-transcriptionally regulated genes detected

The major aim of this study was to show in an objective, large scale, quantitative manner that combining RP and PP measurements (in comparison to each measure independently) is expected to improve the ability to detect meaningful post transcriptional regulation signals. Thus, we focused on objective quantitative measures. Nevertheless, in this section we provide some biological examples related to meaningful/relevant biological cell cycle signals detected by PP and RP. To this end, we will focus on the module inference/clustering analysis performed based on protein-protein interactions among genes detected to be differentially expressed based *both* on PP and RP (90 genes, see Fig. 8C and supplementary table Supplementary_Table_S9_RPiPP_ClusterPEDetails.xlsx). As mentioned, we detected 15 modules (see Fig. 8C); here we will discuss in further detail the four largest modules.

The first module of size 11 genes/proteins includes many genes that encode ribosomal proteins (e.g. RPL3, RPL34, RPS10, RPL35, RPL32, RPL29) which are down regulated (in terms of both RP and PP) in M in comparison to G1. This result supports the hypothesis that translation (specifically the canonical regulatory mechanisms) is globally down regulated during M phase^39^,^40^,^41^ in mammalian cells, and that the down regulation occurs and can be detected also post transcriptionally.

The second module of size 27 genes/proteins includes various M phase specific genes/proteins mainly related to spindle morphogenesis and chromosome movement that are found to be up-regulated based on PP and RP in M phase: for example, one hub in this module is the gene/protein ESPL1 which stabilizes cohesion between sister chromatids before anaphase, and their timely separation during anaphase is critical for chromosome inheritance. Another hub is the gene/protein BUB3 that is involved in spindle checkpoint function, which is up-regulated in M phase together with BUB1. Interestingly the module also includes several kinesins KIF22, KIF20A, KIF18A, KIF23, KIF2C, KIFC1; it was suggested that kinesins and proteins interacting with them are known to have important spindle morphogenesis and chromosome movement in cell division^42^,^43^,^44^,^45^, and our analysis emphasizes their post-transcriptional regulation. Naturally this module also includes (among others) cell cycle regulatory proteins such as CDC20 and CDC8 that are involved in nuclear movement prior to anaphase, chromosome separation, and spindle formation. It also includes various Kinases (e.g. PLK1, CDK1, and TTK) that are involved in regulating the processes mentioned above.

The third module includes 13 genes/proteins related mainly to DNA replication. One hub in this module is the gene FZR1; it is up-regulated in M-phase and is a key regulator of ligase activity of the anaphase promoting complex/cyclosome. The module includes genes/proteins related to DNA replication regulation, and activation and maintenance of the checkpoint mechanisms in the cell cycle that coordinate S phase and mitosis: MCM6, CDC6, MCM3, PCNA, RFC4; all these genes are down regulated (based on RP and PP) at the M-phase as there is no DNA replication during M-phase^46^. The module also includes various genes related to gene expression regulation and proliferation such as the gene DMAP1 which represses transcription and is up-regulated in M-phase. Finally, it includes genes related to cell cycle progression such as the genes CCNA2 and CDK4 which are up-regulated in M-phase.

The fourth module (module number 14) includes 12 genes/proteins which are related among others to dynamic microtubules polymerization, which is an important step of the M-phase^46^. For example, the module’s main hub, TUBB4B (Tubulin, beta 4B class IVb), and 3 additional tubulins (TUBB6, TUBA4A, TUBB4A) are up regulated (according to RP and PP) in M-phase; this fact emphasizes the post transcriptional regulation of microtubules polymerization during M-phase.

To summarize the details depicted above, the genes/proteins detected by RP and PP are highly relevant to cell-cycle biology and teach us about the central role of post transcriptional regulation during the cell cycle.

## Discussion

This study includes the first comparison of RP and PP. We report various analyses that demonstrate that RP and PP can exclusively detect relevant differentially expressed genes. Specifically, based on enrichment and PPI network analyses, we show that genes that are detected by each of these methods, but not by the other, tend to include biologically relevant signals. We evince that the prediction of steady state protein levels can be improved by combining PP and RP measurements. Furthermore, we show that the relevant DE genes detected by each of the methods may have opposite fold-change, demonstrating that the two techniques can detect different aspects of translational regulation, and are thus in part synergistic.

There are three major explanations to the fact that the correlation between 1) a model based on RP, PP, and mRNA and 2) steady state protein levels is not prefect: First, steady state protein levels are a result of many gene expression steps such as the regulation of protein degradation, post-translational regulation, and secretion of proteins. Second, there are different biases in the cases of the various experiments/measurements. For example, the sequencing based experiments have biases related to RNase, while the proteomic based approaches have biases related to protein digestion; in addition, the distribution of protein/peptide length is different in PUNCH-P (where truncated proteins are generated at the first stage) and in steady state protein levels measurements.

Third, some of the differences are due to natural variability among technical repeats and may also be related to the stochasticity (specifically for lowly expressed genes) of the gene expression steps (see, for example,^47^).

We would like to summarize some of the different biases in the RP and PP experiments. The RP major biases can be related to preferences/non-uniform efficiency of the RNase, sequencing biases, inefficiency of the RNase in digesting regions with high ribosome densities, multiple mapping positions of small reads/footprints, ribosomal RNA filtering, biases due biochemical properties/efficiencies/’preference’ of the protocol’s reagents^27^,^38^,^48^,^49^,^50^,^51^.

The PP major biases can be related to preferences/non-uniform efficiency of the protein digestion step, recognition/detection of similar and/or short proteins/peptides, inability to recognize short peptides emerging from ribosomes at the 5′ end of the ORF (the beginning of the translation), mass spectrometry resolution, biases due biochemical properties/efficiencies/’preference’ of the protocol’s reagents^52^,^53^,^54^,^55^,^56^,^57^,^58^.

Thus, naturally, the analyses reported here suggest that one possibility to overcome these problems is via the integration of these techniques. For example, performing both PP and RP experiments should enable detecting more significantly relevant differentially expressed genes than each of the methods separately.

As mentioned above, there are aspects of gene expression not covered by PP, RP, or mRNA levels. These aspects include among others measurements of protein degradation rates, mRNA degradation rates, post-transcriptional regulation, protein secretion and transport. Including these aspects in the regression analysis should improve the correlation with steady state protein levels, and provide better understanding of gene expression regulation. Similar/overlapping analyses that include various (but not all) gene expression steps were performed in very few previous studies^8^,^59^. However, such an analysis has yet to be performed in the cell cycle of mammalian cells (or other organisms) with all the gene expression aspects mentioned above, calling for future studies on this topic.

The Spearman correlations obtained between PP, PP + RP, and PP + RP + mRNA, regression (for RP coverage >0; and on 100 2-fold cross validations, see Methods) with steady state protein levels of G1 are up to 0.681/0.753/0.756 respectively. Similarly, the Spearman correlations obtained between PP, PP + RP, and PP + RP + mRNA, regression with steady state protein levels of M are up to 0.677/0.752/0.757 respectively. The Pearson correlations are similar: correlations between PP, PP + RP, and PP + RP + mRNA, regression with steady state protein levels of G1 are 0.715/0.776/0.781 respectively; correlations between PP, PP + RP, and PP + RP + mRNA and steady state protein levels of M are 0.714/0.775/0.781 respectively.

The mRNA vs. protein levels correlations are comparable or higher than correlations reported in mammals in previous studies based on different techniques and/or tissues/cells: for example, previous studies have generally reported a correlation of up to 0.5–0.64 and sometimes lower between protein and mRNA levels (hence around 30%–40% of the variance of protein levels is explained by mRNA levels^59^,^60^,^61^). Thus, the improved correlations/regressions reported here are significant and high also when comparing to previous studies in the field.

It is important to emphasize that the correlations between RP, PP and regression based on both in mammals have not been reported before. Furthermore, correlations between mRNA and protein levels have not been performed in the HeLa S3 cell-cycle; since post-transcriptional regulation is expected to vary among different tissues, cell-lines, and conditions, we also do not expect to see identical mRNA - protein-levels correlations in all these cases.

The fact that our analyses demonstrate that there are genes detected to be differentially expressed in *opposite* directions via RP and PP, suggests increasing/decreasing ribosomal density detected by RP is not always related to increasing/decreasing ribosomes involved in protein synthesis rates and/or protein abundance. As mentioned, this is possible, for instance, if not only translation initiation but also elongation is regulated during the cell cycle. Specifically, an increase in the initiation rate (which should contribute towards *increasing* ribosome density and RP levels) *together* with an *increase* in the elongation rate in some regions of the mRNA (which should contribute towards *decreasing* ribosome density and traffic jams related to RP levels), may *decrease* the number of ribosomes involved in translation at a certain time point (related to PP and RP levels measurements) even though the protein synthesis rate should clearly *increase*. As aforementioned, the differences between the RP and PP measurements can be related to possible biases in the two techniques.

Finally, the aim of the paper was to show in a simple and clear way that each of the methods PP and RP can detect biological meaningful information not detected by the other method. The various future directions which emerged based on the reported results include: better understanding and modelling of the biases of PP and RP, and developing various novel experimental and computational methods for combining the information detected by the two techniques.

Furthermore, it will be interesting to provide additional biological verifications related to some of the reported results. For example, the genes with RP-PP discrepancy can be studied to better understand the origin of this discrepancy. As mentioned, one explanation may be related to ribosome trafficking/traffic jams/etc, to better understand this issue novel variants of RP, less sensitive to traffic jams need to be developed; they may be based on sequencing of reads related to two or three close ribosomes and not only one ribosome footprint length^38^.

## Methods

### Ribosomal Profiling

#### Ribosomal Profiling Experiment

Two Ribo-Seq experiments (which included parallel RNA-Seq), one with 3 replicates, and the other with one replicate, totalling 4 technical replicates for each phase G1 RP and mRNA, and M RP and mRNA, respectively (all in all 16 samples), were performed. Total mRNA and ribosome-protected fragments were analyzed essentially as described in^51^. In brief, 5 × 106 HeLa S3 cells synchronized to either G1 or M phase were treated with 100 ug/mL cycloheximide for 5 min, harvested on ice and washed twice in ice-cold PBS. Following cell lysis and removal of nuclei by centrifugation, one tenth of the lysate was removed for total mRNA extraction and the rest was digested with 60 units RNase I (Ambion) for 45 min at room temp. Digestion was terminated with SuperaseIN (Ambion) and monosomes were pelleted by ultracentrifugation on a sucrose cushion. Footprint fragments were purified using a 15% polyacrylamide urea gel, and rRNA fragments were removed using Ribo-Zero Magnetic Gold Kit (Epicentre) according to the manufacturer’s instructions. In parallel, mRNA was purified using Oligo (dT) cellulose (Ambion) according to the manufacturer’s instructions and fragmented for 25 min at 94 °C. Both mRNA and ribosome-protected fragments were dephosphorylated with T4 polynucleotide kinase and ligated to Universal miRNA Cloning Linker (NEB). RNA was reverse transcribed using SuperScript III and cDNA was circularized using CircLigase (both Epicentre). Libraries were amplified using indexed Illumina primers, size selected on a 1% agarose gel and extracted using High Pure PCR Purification Kit (Roche).

### Reference Transcriptome Assembly

We decided to compile our reference Human genome based on transcripts seeing as our aim is to quantify known gene mRNA levels and ribosomal footprints and compare them to the PUNCH-P measurements^31^. Annotated (5′UTR, ORF, 3′UTR, chromosome positions) Human transcripts (GRCh37.p11) were downloaded from BioMart^62^. Annotated UTRs were added to the ORFs when available, otherwise flanking 1000nt segments were retrieved from Ensembl GRCh37 release 72 in place of the missing UTRs^63^. In addition, transcripts with UTRs shorter than 4nt were replaced by the respective 1000nt flanking segment. A total of 94895 transcripts were reconstructed, resulting in 20727 unique protein coding genes. There were 13016/94895 annotated 5′UTRs, and 21195/94895 3′UTRs. When UTRs were supplemented from Ensembl and the ORFs differed, we replaced the BioMart ORFs with their Ensembl counterpart, except for cases where the Ensembl ORFs were not composed of whole codons triplets (*i.e.* their length is not divisible by 3), and the BioMart ones were, there were 5884/94895 such cases. This left us with 12547/94895 transcripts not composed of whole codon triplets. In order to rectify this, these transcripts were converted to proteins and compared to the BioMart protein version. If the missing AA was present in the BioMart protein, we tried to uniquely determine the partial codon, by looking at the AA’s synonymous codons. If this was possible the partial codon was completed to a full codon, otherwise an N was added instead, this occurred in 2769/12547 of the transcripts. If the missing AA was not present in the BioMart protein, the ORF was truncated. Transcripts with no stop codon were randomly supplemented with one. BioMart ORFs containing one or two N characters in the beginning were not modified. Considerable specific rRNA contamination may remain even after depletion by subtractive hybridization. Thus, a significant fraction of sequencing reads are derived from digested rRNA present in the monosome sample. Therefore reads mapping to rRNA are first filtered, against a rigorous rRNA database^5^. Aside from rRNA contamination, there are contaminating sequences derived from other abundant ncRNAs, such as tRNAs. The extent of rRNA and ncRNA contamination can vary, particularly when global changes in protein synthesis alter the fraction of active ribosomes, and thus the number of ribosome-protected footprints relative to other RNAs. Thus, reads are also mapped separately to an annotated non-coding RNA database^5^. rRNA (498 genes) and tRNA (452 genes) databases were compiled from BioMart (GRCh37.p11)^62^.

### Mapping Ribosomal Footprints and mRNA Fragments to Transcripts

The following read (ribosomal footprint or mRNA fragment) mapping protocol was devised and implemented, for each of the four replicates separately (for G1 RP and mRNA, M RP and mRNA, totalling 16 replicates):

1. The 3′end adapter CTGTAGGCACCATCAAT was removed from the 51nt long reads using Cutadapt v1.6^64^, retaining only reads with a minimum length of 24nt.
2. These reads were then initially mapped against the rRNA and tRNA databases, using Bowtie v0.12.8^65^: -a –best –strata -n 2 –seedlen 24 –tryhard. In -n mode, alignments may have no more than N mismatches in the seed, which was chosen here to be 2^5^,^48^, with the seed length being 24, as sequencing errors are more likely near the end of the read. Specifying -a instructs bowtie to report all valid alignments, subject to the alignment policy, enabling us to control the mapping selection process, with –best –strata causing bowtie to report only those alignments in the best alignment “stratum”. Throughout the analysis the Bowtie mapping is executed as described. Reads which mapped against the rRNA and tRNA databases were removed.
3. The remaining reads were mapped against the assembled transcriptome using Bowtie as described.
4. Read mapping frequency was determined. The read mapped position is defined to be the read’s 5′ end first nucleotide (changing the definition to 10-20 nt downstream did not change the reported results). Uniquely mapped reads are indentified accordingly. As discussed in^66^, many of the multi-aligned reads are attributable to known duplicated genes and segmental duplications. This is expected for paralogs that are very similar to each other and for internally repeated domains within some genes. If all multi-aligned reads are simply discarded, the end result will be to undercount greatly or even entirely fail to report expression for genes that have closely related paralogs, such as those of the ubiquitin family for example. Specifically in our dataset, the human transcriptome, many of the alternatively spliced transcripts of a gene bear high similarity. Multiple aligned reads were extended to 30nt (the approximated insert length), with a mismatch score calculated. Reads with a single minimal mismatch score were deemed unique. Equal contenders vicinity read density was calculated 30nt upstream and downstream of the mapped read’s 5′ end first nucleotide (the read mapped position). Each of the multiple mapped positions is then assigned a fraction of the read, signifying its relative frequency based on its vicinity read density. In some rare instances the vicinity read density of all the multi-aligned reads is zero (possibly reflecting very recent gene duplication^66^), we then distribute the reads evenly among the mapped positions candidates. The inclusion and proportionate distribution of multiple aligned reads will naturally have variable impact on RNA quantification, with smaller effects on paralogs that are more divergent and larger effects on those that are more similar to each other^66^.

The total number of reads, number of reads mapped to rRNA, number of reads mapped to tRNA, total number of viable reads, number of reads mapped, and number of multi reads, appear in supplementary file Supplementary_Table_S8_RP_PP_Data.xlsx. As mentioned in the main text, the relatively short length of the reads may contribute towards biases in RP as some of the reads can be mapped to many positions in the genome. Our analysis demonstrates that this is the case for on average 8.6% of the reads (among others this may be due to the fact that we map to annotated transcripts and not to the entire genome).

### Ribosome Density and mRNA Levels Quantification

The sensitivity of RNA-Seq in general, and Ribo-Seq in particular, should be a function of both molar concentration and transcript length. Thus transcript levels are quantified in reads per kilobase of exon model per million mapped reads (RPKM). The RPKM measure of read density reflects the molar concentration of a transcript in the starting sample by normalizing for RNA length and for the total read number in the measurement. This facilitates transparent comparison of transcript levels both within and between samples^66^. In ideal conditions where sequenced reads are randomly sampled uniformly from transcripts and no alternative transcripts are derived from an identical genic region, RPKM reflects well the actual transcript abundance levels. In alternatively spliced genes, which comprise 92–94% of human genes^67^,^68^, if ignored, the fact that different isoforms may be of different lengths, results in a “projective normalization” method. It has been shown in a recent paper that the projective normalization method under-estimated the gene expression levels to varying degrees^69^. We quantify RPKM at the gene level, and since reads were mapped to transcripts, the read count was calculated as the sum of the reads mapped to each transcript. We partially correct for the alternative splicing bias by the manner we treat multi-aligned reads as described in the section above. An additional bias is related to ‘correct’ estimation of the mappable gene length based on its transcripts. In order to provide an accurate as possible estimation of the effective gene length, we performed the following procedure: for each gene transcript we extract all the 30 nt sliding windows (approximated insert length), with a slide of 1nt, and calculated the number of unique ones. Thus, reads per KB per million reads (RPKM,^66^), were calculated using the formula:
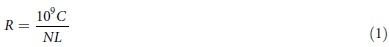
where C is the number of mappable reads that fell onto the gene’s transcripts, N is the total number of mappable reads in the experiment, and L is the estimated gene length in base pairs. For RPKM calculations we considered only the last 40nt of the 5′UTR, entire ORF, and first 40nt of the 3′UTR (these are the regions related to the translation of the main coding region of the transcript). This was performed for each of the 4 replicates separately of M, G1, RP and mRNA. Throughout the analyses we included only genes with >0 ribosomal footprint coverage (though we supply all data in Supplemetary_Table_S8_RP_PP_Data.xlsx).

## PUNCH-P

### PUNCH-P Data

PUNCH-P is a novel system-wide proteomic approach for direct monitoring of translation, termed puromycin-associated nascent chain proteomics, which is based on incorporation of biotinylated puromycin into newly synthesized proteins under cell-free conditions followed by streptavidin affinity purification and liquid chromatography-tandem mass spectrometry analysis, developed by Aviner *et al*.^31^. Aviner *et al*.^31^ generated a global profile of protein synthesis throughout the cell cycle by harvesting thymidine-synchronized HeLa cells at four time points, corresponding to peak S phase, G2/M boundary, mitotic exit, and peak G1 phase, based on fluorescent analysis of DNA content. Synchronization was performed in triplicates, and the samples were processed in parallel for PUNCH-P analysis. The raw intensity data was taken from^31^, consisting of 3 control replicates (nonpuromycylated) and the 4 time points experimental (puromycylated) triplicates (12 in total). Raw MS files were analyzed with MaxQuant software^70^ and the Andromeda search engine^71^. MS/MS were searched against the UniProt human database and an additional list of common contaminants, including avidin. iBAQ and LFQ quantification of the raw intensity data were also obtained from^31^. In order to further eliminate background binders, e.g. ribosomal proteins, the raw intensity data at the four time points were filtered in the following manner:

1. Lowest intensity value imputation to replace missing values, in this case 18^31^, was performed on the experimental (puromycylated) triplicates of each time point and the control (nonpuromycylated) triplicate in preparation for (2).
2. Splitting the data into 2 groups: 3 control samples, and 12 experimental samples, we performed a t-test with 1% FDR^72^, filtering out non-significant experimental data points.
3. The non-significant experimental data points identified in (2) were also removed from the iBAQ and LFQ version.
4. The experimental data was further filtered for a minimum of proteins detected in at least 2 of the 3 replicates. This was performed separately for the LFQ and iBAQ versions. This was performed in order to ensure that the protein levels reported are not a result of noise, a minimum of 2 replicates out of the 3 must show protein detection to be considered significant (this is a common filtering approach in the field). We tested variations of this filtering procedure and received similar results, but since the authors of^31^ employed this filtering method we decided to be consistent with that.
5. Averaging of the per time point triplicates was performed separately for the LFQ and iBAQ versions.

A total of 5105 proteins were identified in at least two of three samples, of which 4984 were specific to the puromycylated samples relative to nonpuromycylated controls (we supply all the PP replicates in iBAQ and LFQ form in Supplementary_Table_S8_RP_PP_Data.xlsx so the reader can manipulate them to his needs).

### Steady State Protein Levels

Estimation of steady state protein levels for the G1 and M phases were taken from^31^. It is important to emphasize that mass spectrometry (PP and PSS) are highly quantitative, but not in an absolute sense. Since we expected a monotone relation between protein levels and RP or PP we performed Spearman correlation analysis.

The experimental data was filtered for a minimum of proteins detected in at least 2 of the 3 replicates (we tested variations of this filtering procedure and received similar results). This was performed separately for the LFQ, and iBAQ versions (we supply all the PSS replicates in iBAQ and LFQ form in Supplementary_Table_S8_RP_PP_Data.xlsx so the reader can manipulate them to his needs).

### HeLa Cells for All Experimental Procedures

PSS, RP, PP, and mRNA, were obtained from the same batch of cells but not simultaneously.

### Determining Differentially Expressed Genes in M and G1

Differentially expressed genes between M and G1 Ribo-Seq measurements were calculated according to^36^, a method based on the negative binomial distribution, with variance and mean linked by local regression (see Supplementary Methods for further details), where the top 10% most significant FDR p-values were selected. Since differential expression is determined at the gene level, and since reads were mapped to transcripts, the read count was calculated as the sum of the reads mapped to each transcript. Moreover, we considered only the last 40nt of the 5′UTR, entire ORF, and first 40nt of the 3′UTR (corresponding to the main coding region of the gene). For PUNCH-P differentially expressed genes between M and G1 are determined according to highest significant (ANOVA) fold-change. The top 10% highest significant fold change was selected. Results for both groups are robust to reasonable variations in this number.

The analysis performed to detect differentially expressed genes differs between Rib-Seq and PUNCH-P due to the nature of the data. For example, Ribo-Seq (like RNA-Seq in general) is assumed to have a negative binomial distribution (see Supplementary Methods), while proteomic measurements such as PUNCH-P are (usually) analyzed with ANOVA^31^,^73^. To show that employing the same statistical method to both approaches is inaccurate, we performed the following analysis: We calculated Ribo-Seq differentially expressed (DE) genes according to the methodology used for detecting DE in PUNCH-P, and compared them to the DE genes as previously detected for Ribo-Seq (namely DESeq). Now both selected group sizes are the same (1215), and the intersection was 76, for which we calculated a hyper-geometric p-value, and received p = 1 (i.e. the overlap between the two approaches is not significant which does not make sense). Conversely the p-value achieved for the intersection of Ribo-Seq DE and PUNCH-P DE as performed in the paper was p =3.3·10^-51^.

### Correlations and Regression Analysis

In the correlation analysis the M, G1, RP and mRNA vectors were the result of averaging across the respective 4 replicates RPKM (after normalizing each replicate by its mean, and the resultant averaged vector is multiplied by the average of the replicate means), in order to minimize experimental noise. The PP M and G1 vectors were the result of averaging across the 3 replicates after the filtering described above. All correlations reported in this study are Spearman. We performed a 2-fold cross validation 100 times per Spearman linear regressor. The regression analysis was performed as a function of the coverage for the following 7 RP coverage groups: >0, ≥10%, ≥20%, ≥30%, ≥40%, ≥50%, ≥60%. The coverage is related to the expression levels of the genes (lower coverage corresponds to lower expression levels) and to the expected bias in the RP protocol (lower coverage corresponds to higher expected bias).

Note that we believe that Spearman (and not Pearson) correlations are the correct way to evaluate the reported relations since we do not expect them to be linear due to the following reasons: 1. Due to translation regulation (and other post transcriptional regulatory steps such as degradation of mRNAs and proteins) the protein levels are not expected to be proportional to the mRNA levels. 2. Due to the limitations of the approaches often the relation between the variable and its measurements is not linear but saturates at higher levels.

### Various data utilized in the analysis

#### Pathways

Human pathways data was downloaded from^74^. Since the flatfile (http://wikipathways.org/wpi/cache/wikipathways_data_Homo%20sapiens.tab, accessed on the 30/05/2014) contained only a subset of the genes reported in the individual pathway pages (such as http://www.wikipathways.org/index.php/Pathway:WP100, accessed on the 30/05/2014), we parsed the individual pathway pages listed in the flatfile. Specifically, each pathway in the flatfile has its relevant url listed, for example for ‘Glutathione metabolism’ the url is http://www.wikipathways.org/index.php/Pathway:WP100. We programmatically retrieved each url page, and according to the html tags of the page identified the pathway genes, those whose IDs are not ENSG, namely Uniprot, Entrez, and Affymetrix IDs, were then converted to ENSG accordingly. Our parsed pathways can be found in supplementary file Supplementary_Table_S2_Human_Pathways.xlsx. In the subsequent analyses we only included pathways with seven or more genes (194/372).

#### Pathway Enrichment Score

A pathway enrichment score for each pathway is calculated according to a hypergeometric p-value in terms of the number of differentially expressed genes (calculated as defined above) in ribosomal density (RP) excluding PUNCH-P (PP): RP-PP, in PP-RP, and the intersection RP∩PP, showing that each technique enables detecting biological meaningful signals that are not detectable by the second technique.

#### DAVID Analysis

DAVID functional annotation analyses were performed using the DAVID Web Service (DAVID-WS)^75^. The background list in each analysis was the 20,727 unique protein coding genes described above. The categories utilized were: BBID, BIOCARTA, COG_ONTOLOGY, GOTERM_BP_FAT, GOTERM_CC_FAT, GOTERM_MF_FAT, INTERPRO, KEGG_PATHWAY, OMIM_DISEASE, PIR_SUPERFAMILY, SMART, SP_PIR_KEYWORDS, UP_SEQ_FEATURE, GENERIF_SUMMARY, DIP, altogether 15. Term clustering analysis was performed with the following parameters: overlap = 3; initialSeed = 3; finalSeed = 3; linkage = 0.5; kappa = 50. Gene cluster analysis was performed with the following parameters: overlap = 4; initialSeed = 4; finalSeed = 4; linkage = 0.5; kappa = 35. Each representing the medium stringency. Additional information regarding the DAVID analysis can be found in the Supplementary Methods.

#### Protein-Protein Interactions

PPI data was taken from^76^, which contains 372766 molecular interactions for over 30000 human proteins, which are mapped to 17694 unique genes. Besides protein–protein interactions from 12 different resources (including HPRD^77^, BioGrid^78^, IntAct^79^, DIP^80^, BIND^81^ and Reactome^82^ databases; as well as four interaction maps produced by computational predictions^83^,^84^,^85^,^86^ and two high-throughput yeast-2-hybrid (Y2H) screens^87^,^88^), UniHI 7 also comprises curated transcriptional regulatory interactions from three complementary databases TRANSFAC^89^, miRTarBase^90^ and HTRIdb^91^. Since UniHI 7 is in Entrez gene ID format, we converted to Ensembl gene Ids using: HGNC^92^, Ensembl BioMart^62^, DAVID^75^, UniProt^93^, IDconverter^94^ and g:Profiler^95^. Of the 17694 genes in the PPI data, we managed to convert 16720, resulting in 357019/372766 interactions.

The nodes in the resultant PPI network, which is an undirected graph with 4271 connected components, with the main connected component the size of 16445 nodes and 357007 edges (interactions), while the rest are single nodes or an edge (12), thus we focused on the main connected component, which we will refer to as the PPI network.

Two PPI analyses were performed, with differentially expressed (DE) genes according to Ribo-Seq (RP) and PUNCH-P (PP) determined as described above:

Three PPI network colouring schemes were defined where black nodes represented DE genes: 1. RP-PP (999 genes). 2. PP-RP (203 genes). 3. RP∩PP (125 genes), respectively. We computed the mean distance (md) in each between all black nodes. For each case we compute a PPI empirical p-value by randomizing each PPI network 100 times respectively, while maintaining the degree distribution of the network, and calculating the black node distance. Shorter distances between DE PPI nodes means more meaningful biological signals (we expected to see physical interactions between DE genes). This analysis shows that each of the techniques uncovers significant protein-protein interactions that cannot be detected by the other.

We performed a clustering analysis (Newman algorithm^37^), on the protein-protein interaction network using the previously described differentially expressed genes according to Ribo-Seq (RP) and PUNCH-P (PP) respectively, divided into the following four groups: 1. RP-PP. 2. PP-RP. 3. RP∩PP (See Supplementary for RP∪PP). We performed pathway enrichment analysis on the resultant clusters. The Newman algorithm optimizes the quality function known as “modularity” over the possible divisions of a network, expressing modularity in terms of the eigenvectors of a characteristic matrix for the network (called the modularity matrix), and this expression leads to a spectral algorithm for community detection. We visualized our clustering analysis using Cytoscape^96^.

## Additional Information

**How to cite this article**: Zur, H. *et al*. Complementary Post Transcriptional Regulatory Information is Detected by PUNCH-P and Ribosome Profiling. *Sci. Rep.* **6**, 21635; doi: 10.1038/srep21635 (2016).

## Supplementary Material

Supplementary Information

Supplementary Table 1

Supplementary Table 2

Supplementary Table 3

Supplementary Table 4

Supplementary Table 5

Supplementary Table 6

Supplementary Table 7

Supplementary Table 8

Supplementary Table 9

## Figures and Tables

**Figure 1:**
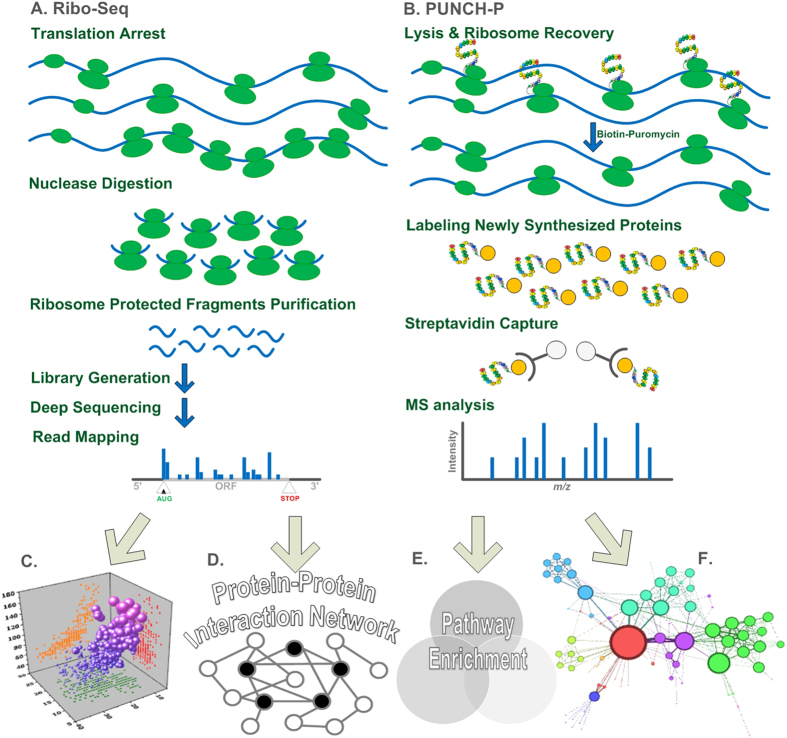
A schematic illustration of the study: Ribo-Seq (**A**) and PUNCH-P (**B**) data are used simultaneously in-order to augment the information which can be extracted based on each alone (description of the methods appears in the main text). (**C**) Predictive power of steady-state protein levels based on the two approaches are assessed. (**D**) Protein-protein interaction (PPI) analyses are performed based on differentially expressed (DE) genes in the cell-cycle phases M and G1 based on each approach. (**E**) Pathway enrichment analyses are performed based on the DE genes detected based on each approach. (**F**) Clustering of the PPI sub-networks induced by DE genes detected based on each approach is performed.
