# Supplementary material for: Complementary Post Transcriptional Regulatory Information is Detected by PUNCH-P and Ribosome Profiling
Source: Sci Rep. 2016 Feb 22;6:21635. doi: 10.1038/srep21635 (PMC4761937; doi:10.1038/srep21635)
Supplement: Supplementary Information [file srep21635-s1.pdf]

Supplementary of 'Complementary Post Transcriptional Regulatory Information is Detected by PUNCH-P and Ribosome Profiling'

Hadas Zur*[,1], Ranen Aviner*[,2], Tamir Tuller[1,3]

[1]Department of Biomedical Engineering, the Engineering Faculty, Tel Aviv University. [2]Department of Cell Research and Immunology, Tel-Aviv 69978, Israel

[3]The Sagol School of Neuroscience, Tel Aviv University, Tel Aviv University.

***Equal contribution**

Contact: tamirtul@post.tau.ac.il (TT)

1 Supplementary Information Contents 3
2 Methods 4
2.1 FACS analysis of the synchronized cells 4
2.2 Ribosomal profiling experiment replicates 4
2.3 Determining differentially expressed genes from Ribo-Seq 5
2.4 DAVID analysis 6
3 Results 7
3.1 PSS Correlation Analysis with mRNA 7
3.2 Pathway Enrichment Analysis 7
3.3 Modules of differentially post-transcriptionally expressed genes and physical interactions 11
3.4 Genes detected to be *oppositely* regulated based on the different methods 11
3.5 The reported results cannot trivially be explained by biological and technical variability within each procedure 14
3.6 RP, PP, and mRNA Pearson correlation with PSS 16
3.7 Supplementary Tables Description 18
4 References 19

1 Supplementary Information Contents

The supplementary is organized as follows and contains the following information:

2. Supplementary Methods contains:
2.1 Demonstration that the cell cycle arrest was efficient.
2.2 The correlations between the four respective replicates of the Ribo-Seq and RNA-Seq experiments per cell cycle phase.
2.3 Details regarding how Ribo-Seq differentially expressed genes were determined.
2.4 Details regarding the DAVID analysis.
3. Supplementary Results contains:
3.1 Spearman correlations between PSS and mRNA levels.
3.2 The full results of the pathway enrichment analysis. Illustrative examples of genes similarly regulated according to both approaches.
3.3 To better understand the differentially expressed genes detected by PP and RP we performed a clustering analysis (Newman algorithm [1], see main text Methods) on the PPI network, here we show the results for the RP∪PP group.
3.4 The full results of pathway enrichment for genes detected to be regulated in the opposite direction according to Ribo-Seq and PUNCH-P. Illustrative examples of genes from each of the oppositely regulated 2 groups.
3.5 A section explaining why the reported results cannot trivially be explained by biological and technical variability within each procedure.
3.6 Pearson correlations for RP, PP, and mRNA levels with steady state protein levels.
3.7 A description of the paper’s 9 supplementary tables.

2 Methods

2.1 FACS analysis of the synchronized cells

The figure below includes the cell count and DNA content for the G1 and M conditions, demonstrating that the cell cycle arrest was efficient.

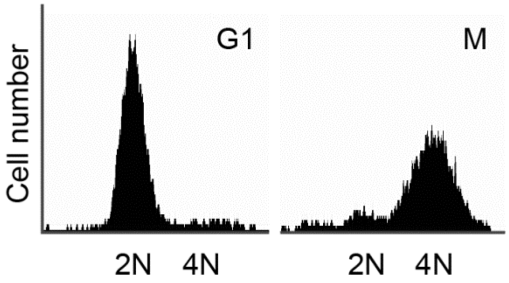

Figure 1: Cell synchronization: Cell count (y axis) and DNA content (x axis) for G1 and M conditions.

2.2 Ribosomal profiling experiment replicates

Two ribosomal profiling experiments, which measured mRNA levels in parallel, were conducted, one with 3 replicates (rep1, rep2, rep3), and one with 1 replicate (rep4). As can be seen in Figure 1 and 2, the Spearman correlation between the replicates is significantly high, and the results of all analyses performed in the paper are robust to utilizing individual replicates .

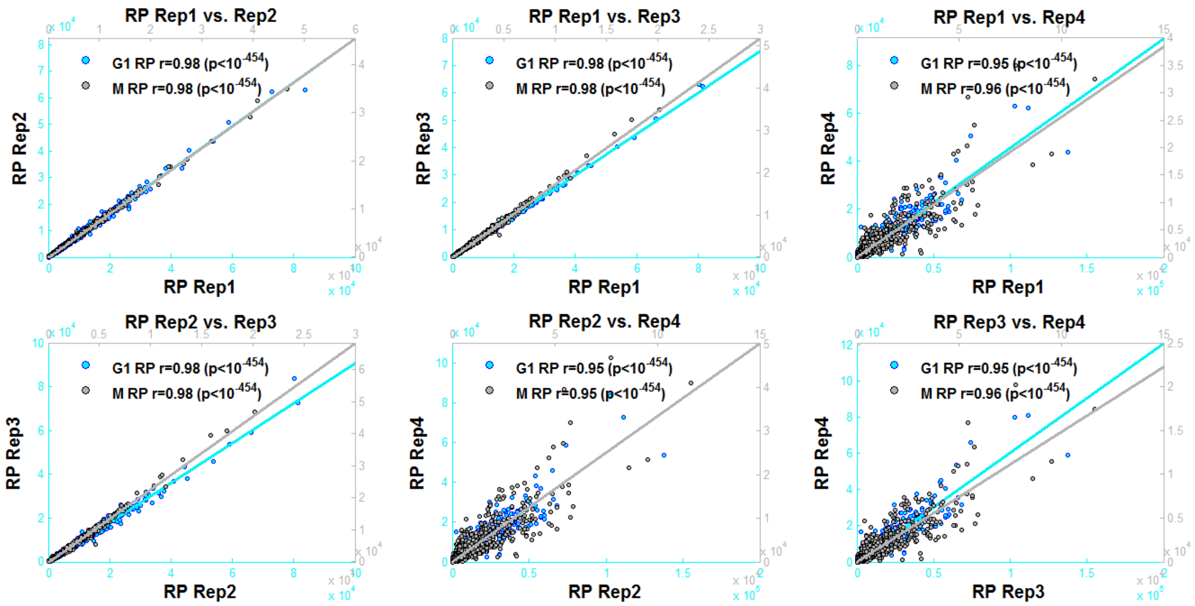

Figure 2: Spearman correlation results of the 4 ribosomal profiling replicates

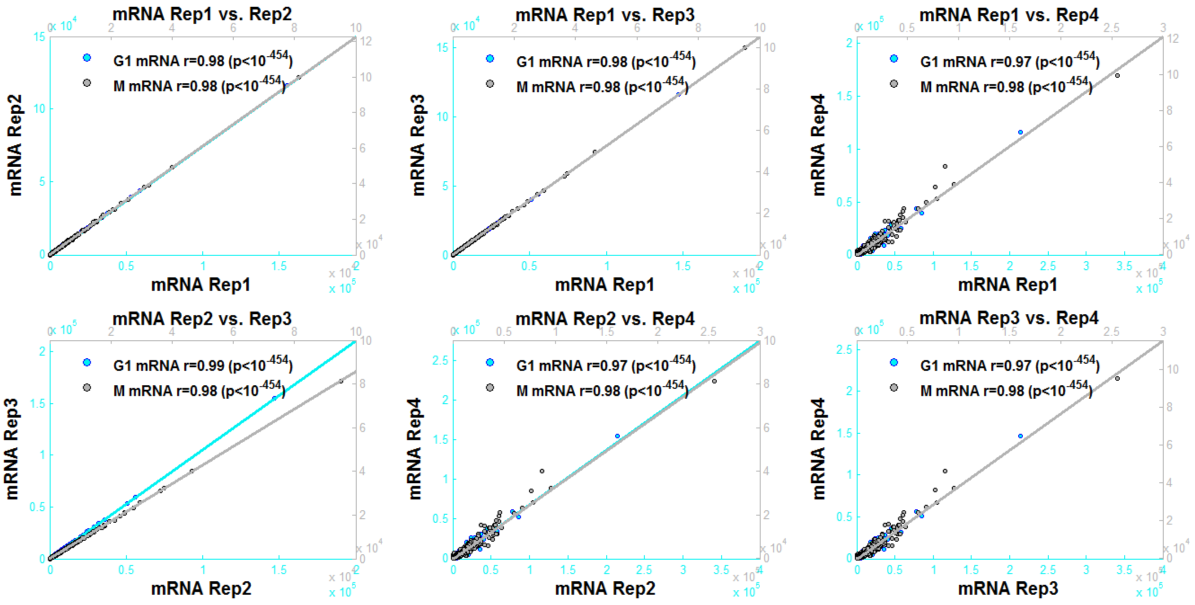

Figure 3: Spearman correlation results of the 4 mRNA levels replicates

2.3 Determining differentially expressed genes from Ribo-Seq

Differentially expressed genes between M and G1 are calculated according to [2], a method called DESeq based on the negative binomial distribution, with variance and mean linked by local regression. Briefly, Anders *et al.* [2] devised a statistical test to decide whether, for a given gene, an observed difference in read counts is significant, that is, whether it is greater than what would be expected just due to natural random variation. If reads were independently sampled from a population with given, fixed fractions of genes, the read counts would follow a multinomial distribution, which can be approximated by the Poisson distribution [3, 4]. However, it has been noted that the assumption of Poisson distribution is too restrictive [5, 6]: it predicts smaller variations than what is seen in the data. Therefore, the resulting statistical test does not control type-I error (the probability of false discoveries). To address this so-called overdispersion problem, it has been proposed to model count data with negative binomial (NB) distributions [7], with parameters uniquely determined by mean μ and variance σ^2, and this approach is used in the *edgeR* package for analysis of SAGE and RNA-Seq [6, 8]. However, the number of replicates in data sets of interest is often too small to estimate both parameters, mean and variance, reliably for each gene. For *edgeR*, Robinson and Smyth assumed [9] that mean and variance are related by $\sigma^2 = \mu + \alpha\mu^2$, with a single proportionality constant α that is the same throughout the experiment and that can be estimated from the data. Hence, only one parameter needs to be estimated for each gene, allowing application to experiments with small numbers of replicates. Anders *et al.* extend this model by allowing more general, data-driven relationships of variance and mean, provide an effective algorithm for fitting the model to data, and show that it provides better fits. As a result, more balanced selection of differentially expressed genes throughout the dynamic range of the data can be obtained.

DESeq has three sets of parameters that need to be estimated from the data:

1. Library size parameters.

2. Gene abundance parameters under each experimental condition.

3. The smooth functions that model the dependence of the raw variance on the expected mean.

Estimating Library Size Factor

The expectation values of all gene counts from a sample are proportional to the sample's library size. The effective library size can be estimated from the count data. Compute the geometric mean of the gene counts across all samples in the experiment as a pseudo-reference sample. Each library size parameter is computed as the median of the ratio of the sample's counts to those of the pseudo-reference sample. The counts can be transformed to a common scale using size factor adjustment.

Estimate the gene abundance

To estimate the gene abundance for each experimental condition you use the average of the counts from the samples transformed to the common scale (Eq. 6 in [2]).

Estimating Negative Binomial Distribution Parameters

In the model, the variances of the counts of a gene are considered as the sum of a shot noise term and a raw variance term. The shot noise term is the mean counts of the gene, while the raw variance can be predicted from the mean, i.e., genes with a similar expression level have similar variance across the replicates (samples of the same biological condition). A smooth function that models the dependence of the raw variance on the mean is obtained by fitting the sample mean and variance within replicates for each gene using local regression function. Sample variances transformed to the common scale are calculated according to Eq. 7 in [2], while the shot noise term is estimated according to Eq. 8 in [2], and the sample variance is calculated by adding the shot noise bias term to the raw variance according to Eq.9 in [2].

Testing for Differential Expression

Having estimated the mean-variance dependence, one can test for differentially expressed genes between the samples. Define, as test statistic, the total counts in each condition, and their overall sum. Parameters of the new negative binomial distributions for the count sums can be calculated according to Eqs. 12-14 in [2], and the numerical calculation of the p-values for the statistical significance of the change between the experimental conditions (differential expression) is detailed in Eq. 11. The p-values are empirically adjusted from the multiple tests for false discovery rate (FDR) with the Benjamini-Hochberg procedure [10].

[See the Matlab tutorial: http://www.mathworks.com/help/bioinfo/examples/identifying-differentially-expressed-genes-from-rna-seq-data.html].

2.4 DAVID analysis

We added the following to the DAVID defaults:
Literature: GENERIF_SUMMARY
Protein_Interactions: DIP.
We define our entire gene set as background and generate a Chart Report, that is an annotation-term-focused view which lists annotation terms and their associated genes under study.

DAVID EASE Score Threshold (Maximum Probability):

The threshold of EASE Score, a modified Fisher Exact P-Value, for gene-enrichment analysis. It ranges from 0 to 1. Fisher Exact P-Value = 0 represents perfect enrichment. Usually P-Value is equal or smaller than 0.05 to be considered strongly enriched in the annotation categories.

3 Results

3.1 PSS Correlation Analysis with mRNA

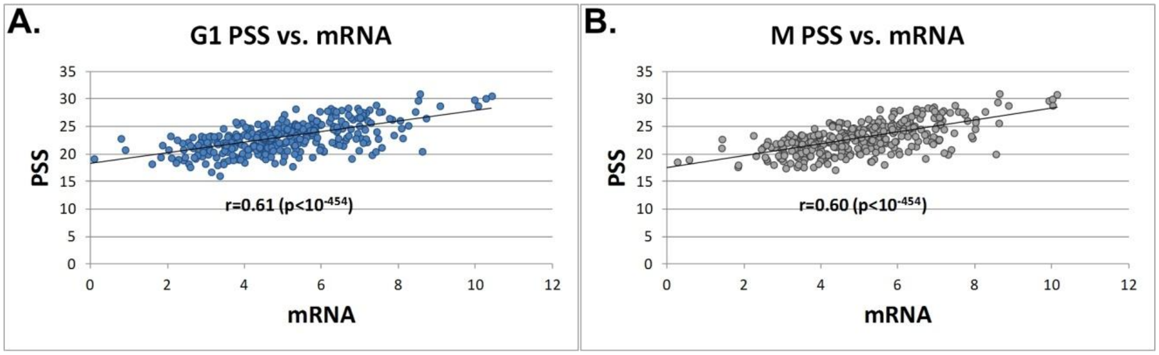

Figure 4: Scatter plot of steady state protein levels (PSS) (y-axis , data is log2-scaled) and mRNA levels (x-axis, read count log2-scaled RPKM (see Methods)) G1 phase. B. Scatter plot of PSS (y-axis log2(intensity)) and mRNA levels (x-axis, read count log2-scaled RPKM (see Methods)) M phase. Reported correlations are Spearman.

3.2 Pathway Enrichment Analysis

Table 1: Full list of significantly enriched pathways according to differentially expressed genes in PUNCH-P (PP) and Ribo-Seq (RP) organized according to: 1. RP-PP (RP DE genes excluding overlapping PP genes). 2. PP-RP (PP DE genes excluding overlapping RP genes). 3. RP∩PP (the intersection of DE RP and PP genes).

RP-PP	p	RP∩PP	p	PP-RP	p
Translation Factors	6.6e-09	Electron Transport Chain	5.2e-03	Matrix Metalloproteinases	4.3e-03
Electron Transport Chain	8.2e-19	Cell cycle	1.9e-13	AMPK signalling	4.1e-02
Androgen receptor signalling pathway	2.8e-04	Integrated Cancer pathway	1.7e-03	Selenium Pathway	1.6e-02
Selenium Pathway	3.8e-02	Integrated Breast Cancer Pathway	2.6e-02	miRNA regulation of DNA Damage Response	4.6e-02
miRNA regulation of DNA Damage Response	3.8e-04	Apoptosis Modulation by HSP70	7.7e-03	Vitamin B12 Metabolism	2.2e-02
Cell cycle	3.8e-02	EGF/EGFR Signaling Pathway	2.5e-02	Energy Metabolism	1.8e-03
Proteasome Degradation	4.6e-13	G1 to S cell cycle control	1.1e-04	Folate Metabolism	4.9e-02

SREBP signalling	1.5e-02	DNA Replication	8.3e-06	Cell cycle	1.5e-05
Integrated Breast Cancer Pathway	3.5e-02	Cytoplasmic Ribosomal Proteins	3.8e-05	SREBP signalling	5.7e-03
TNF alpha signalling Pathway	3.5e-02			Cell Differentiation – meta	1.4e-03
Keap1-Nrf2 Pathway	4.9e-02			Cell Differentiation – Index	5.5e-04
Focal Adhesion	2.4e-03			Adipogenesis	1.2e-04
Signalling of Hepatocyte Growth Factor Receptor	1.4e-02			TGF beta Signalling Pathway	5.2e-03
TGF beta Signalling Pathway	5.9e-03			Oxidative Stress	4.0e-02
MAPK signalling pathway	2.5e-03			G1 to S cell cycle control	1.0e-05
Nucleotide Metabolism	3.0e-02			DNA Replication	1.0e-03
Eukaryotic Transcription Initiation	2.5e-05			TGF Beta Signalling Pathway	2.1e-02
Oxidative Stress	2.8e-02			DNA damage response	4.0e-02
TGF Beta Signalling Pathway	2.1e-02			Prostaglandin Synthesis and Regulation	5.0e-03
DNA damage response	4.0e-02				
Prostaglandin Synthesis and Regulation	5.0e-03				
TGF Beta Signalling Pathway	2.1e-02				
DNA damage response	4.0e-02				
G13 Signaling Pathway	1.4e-02				
Senescence and Autophagy	6.5e-03				
Oxidative phosphorylation	2.1e-09				
DNA damage response	2.4e-03				

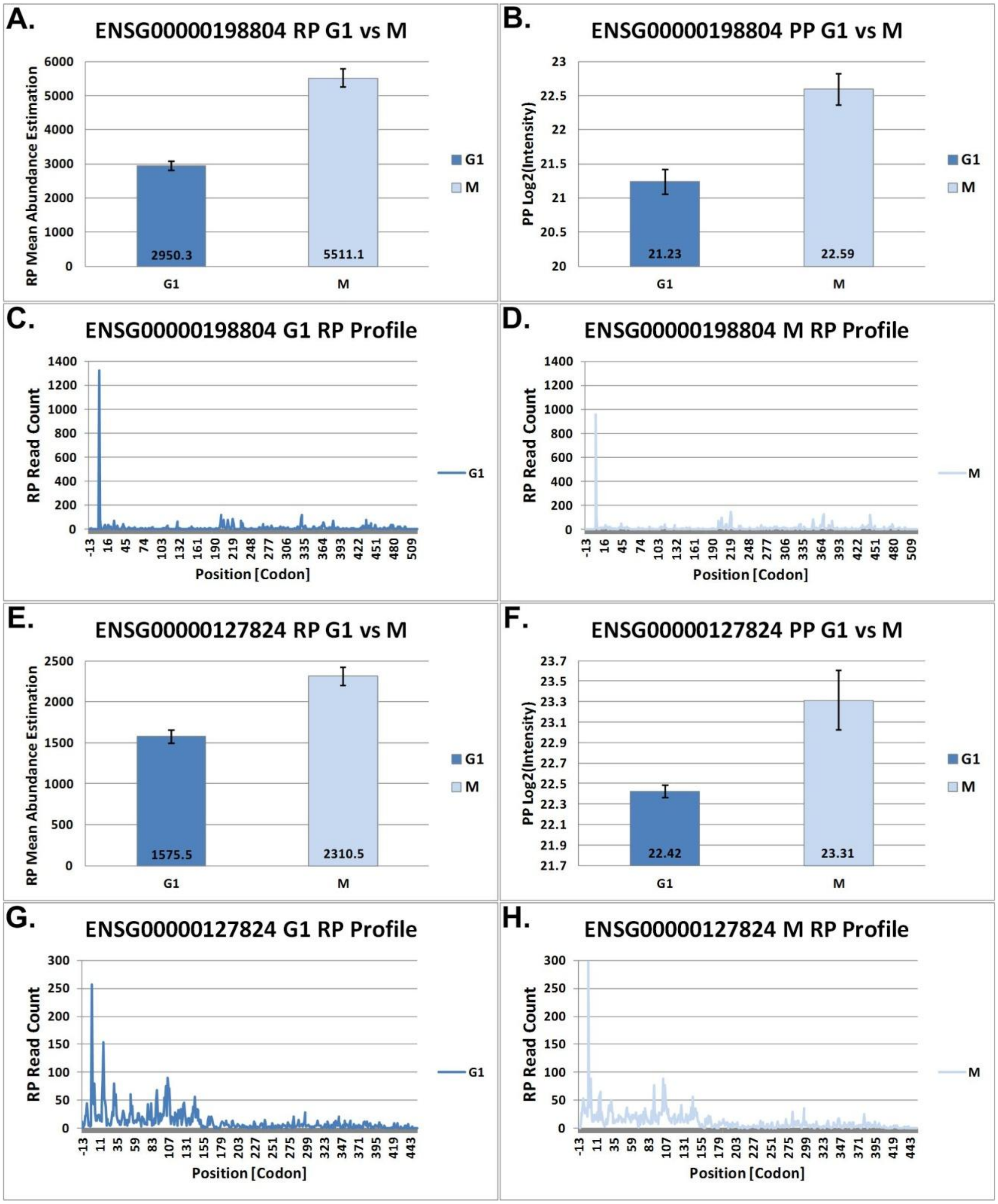

Figure 5: An example of 2 genes upregulated in M phase as compared to G1 according to both Ribo-Seq (RP) and PUNCH-P (PP). Each gene has four panels: A.,E. The mean RP abundance estimation according to DESeq [2]. B.,F. The PP log2(intensity). C.,G. The G1 RP per codon read count profile summed across the 4 replicates. D.,H. The G1 RP per codon read count profile summed across the 4 replicates.

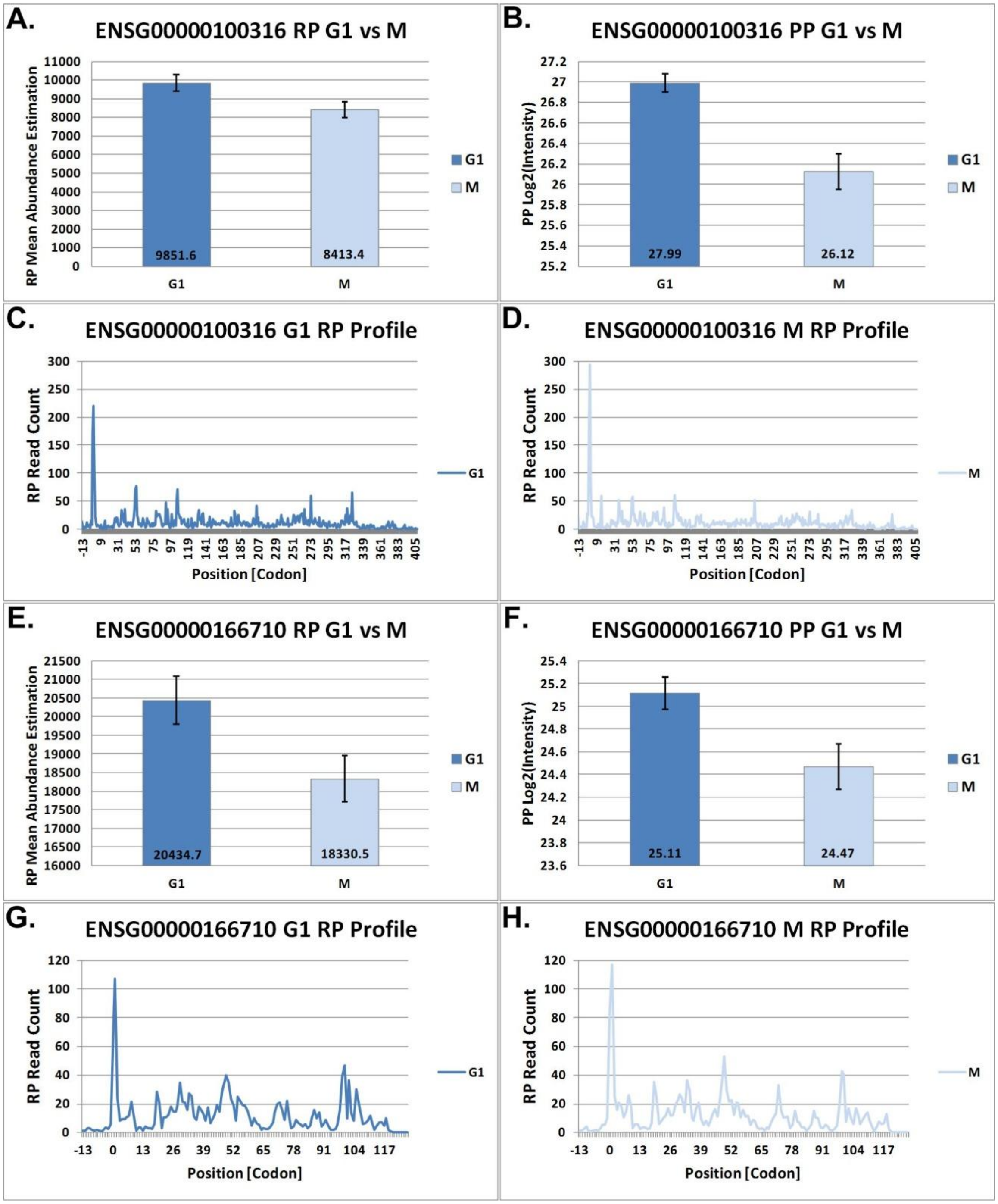

Figure 6: An example of 2 genes downregulated in M phase as compared to G1 according to both Ribo-Seq (RP) and PUNCH-P (PP). Each gene has four panels: A.,E. The mean RP abundance estimation according to DESeq [2]. B.,F. The PP log2(intensity). C.,G. The G1 RP per codon read count profile summed across the 4 replicates. D.,H. The G1 RP per codon read count profile summed across the 4 replicates.

3.3 Modules of differentially post-transcriptionally expressed genes and physical interactions

We performed a clustering analysis (Newman algorithm [30], see methods), on the protein-protein interactions network using the previously described differentially expressed genes according to Ribo-Seq (RP) and PUNCH-P (PP) respectively.

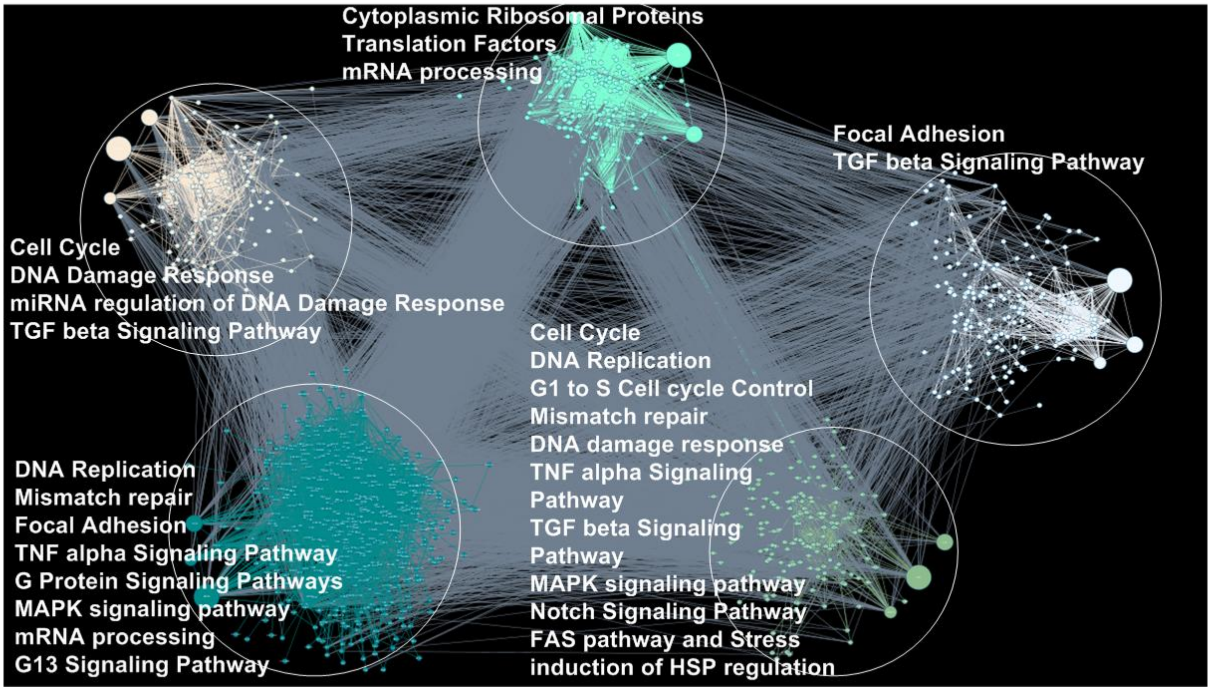

Figure 7: RP∪PP clusters: 1168 genes participate, resulting in 5 clusters. For the full cluster pathway enrichment see Supplementary_Table_S6_ClusterPathwayEnrichment.xlsx.

3.4 Genes detected to be *oppositely* regulated based on the different methods

Table 2: Differentially expressed genes according to both RP and PP M/G1 fold-change but in opposite directions were utilized to perform pathway enrichment (we report significant and borderline significant pathways).

RP > 0 & PP < 0	p	RP < 0 & PP > 0	p
**AMPK signaling**	1.2e-05	SREBP signaling	2.5e-02
SREBP signalling	8.7e-06	Squamous cell TarBase	9.4e-05
Squamous cell TarBase	1.6e-02		
Fatty Acid Biosynthesis	3.7e-03	**G Protein Signaling Pathways**	5.0e-03
**G1 to S cell cycle control**	3.5e-02	Glycogen Metabolism	2.9e-07
**DNA Replication**	2.7e-05	**G13 Signaling Pathway**	5.6e-07
**Cell Cycle**	6.9e-02	mRNA processing	8.0e-02
ID Signaling Pathway	7.3e-02		
**Integrin-mediated cell adhesion**	6.7e-02		

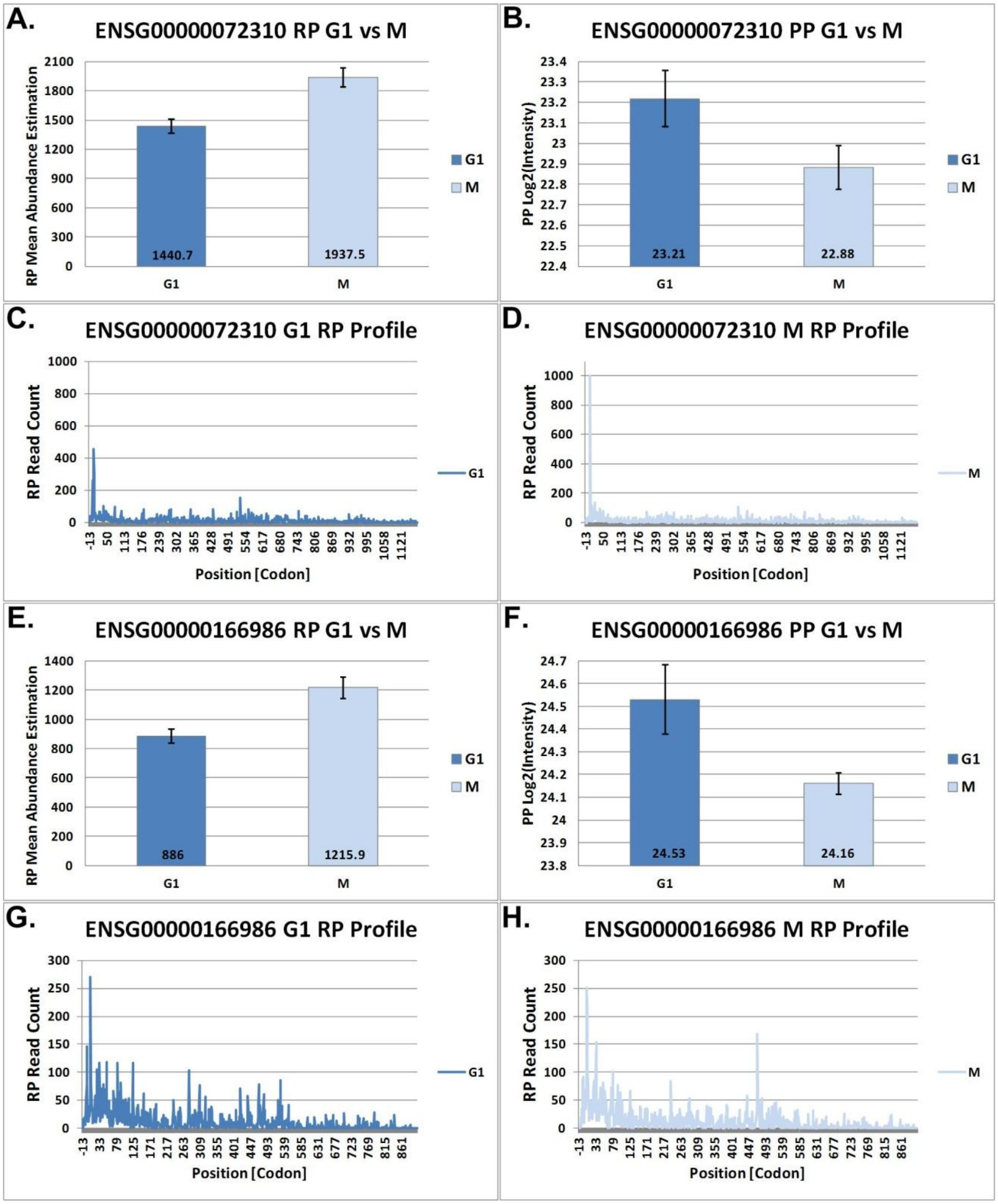

Figure 8: An example of 4 genes upregulated in M phase as compared to G1 according to Ribo-Seq (RP) and downregulated in M as compared to G1 according to PUNCH-P (PP). Each gene has four panels: A.,E. The mean RP abundance estimation according to DESeq [2]. B.,F. The PP log2(intensity). C.,G. The G1 RP per codon read count profile summed across the 4 replicates. D.,H. The G1 RP per codon read count profile summed across the 4 replicates.

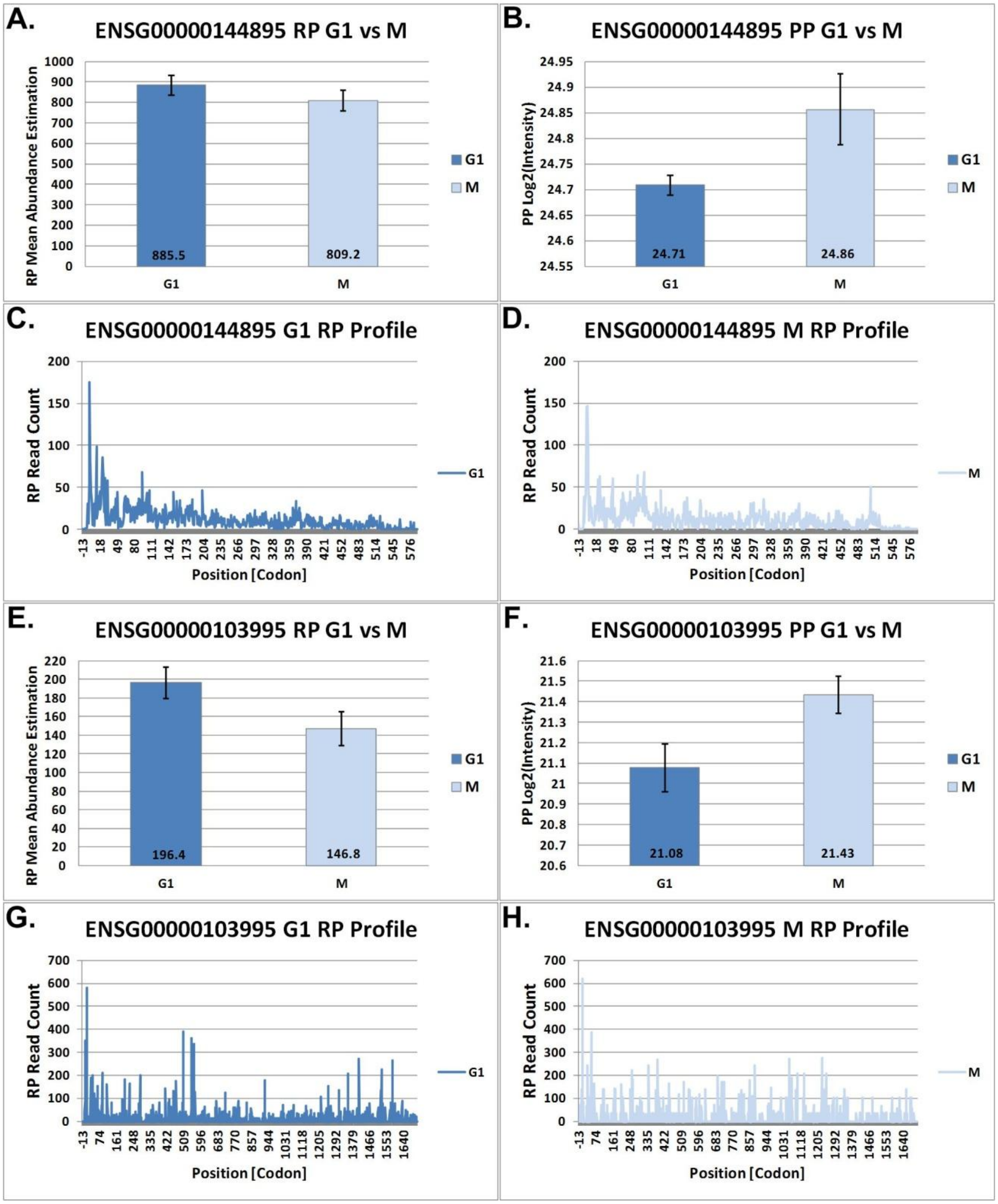

Figure 9: An example of 4 genes downregulated in M phase as compared to G1 according to Ribo-Seq (RP) and upregulated in M as compared to G1 according to PUNCH-P (PP). Each gene has four panels: A.,E. The mean RP abundance estimation according to DESeq [2]. B.,F. The PP log2(intensity). C.,G. The G1 RP per codon read count profile summed across the 4 replicates. D.,H. The G1 RP per codon read count profile summed across the 4 replicates.

3.5 The reported results cannot trivially be explained by biological and technical variability within each procedure

To demonstrate that the reported results cannot be explained by technical variability within each procedure, i.e. to show that the improved prediction of PSS when adding RP (and mRNA) to PP (and vice versa) is not due to any randomness that occurs among different technical repeats, but due to additional/”orthogonal” “information” provided by RP, instead of testing the regressors *PP*, *PP+RP* (based on the average across the four replicates) *(PP+RP+mRNA)* depicted in Figure 5, we tested the regressors of all combinations of the four RP replicates *RPi*, *RPi+RPj*, *RPi+RPj+mRNA*, and showed that the correlation with *PSS* (and improvement in correlation with the addition of variables) is lower. We performed the following analyses:

Utilizing the four RP replicates from the two ribosomal profiling experiments, one with 3 replicates (Rep1, Rep2, Rep3), and one with 1 replicate (Rep4), as described above, we performed the regressor analysis illustrated in Figure 5 of the main text, only now replacing the PP and averaged RP (across the 4 replicates) measurements by all replicate pairs (see Supplementary Figure 2), for RP coverage > 0. As can be seen, while the regressors based on both PP and RP achieve a steady increase in the correlations with steady state protein levels (PSS), the regressor based only on RP replicates plateaus.

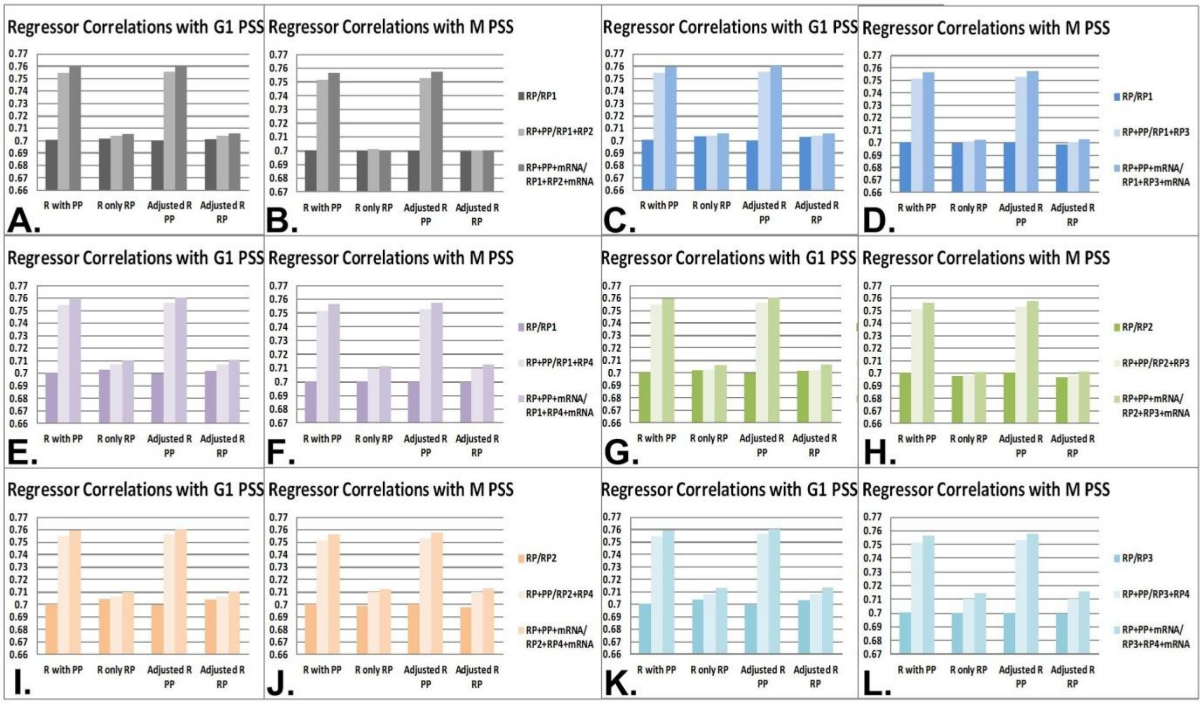

Figure 10: For every RP replicate pair, we compared the correlation results achieved by combining the averaged RP and PP measurements for: G1 PSS regressor results: averaged RP (r=0.701, $p<10^{-454}$), averaged RP and PP ((r=0.755, $p=4.5\cdot10^{-311}$)), averaged RP, PP and mRNA (r=0.759, $p=9.3\cdot10^{-320}$); and M PSS regressor results: averaged RP (r=0.701, $p<10^{-454}$), averaged RP and PP ((r=0.751, $p=3.3\cdot10^{-307}$)), averaged RP, PP and mRNA (r=0.756, $p=2.2\cdot10^{-319}$), respectively, with: A. G1 PSS regressor results: RP1 (r=0.702, $p<10^{-454}$), RP1 and RP2 ((r=0.704, $p<10^{-454}$)), RP1, RP2 and mRNA (r=0.705, $p<10^{-454}$). B. M PSS regressor results: RP1 (r=0.70, $p<10^{-454}$), RP1 and RP2 ((r=0.701, $p<10^{-454}$)), RP1, RP2 and mRNA (r=0.701, $p<10^{-454}$). C. G1 PSS regressor results: RP1 (r=0.704, $p<10^{-454}$), RP1 and RP3 ((r=0.704, $p<10^{-454}$)), RP1, RP3 and mRNA (r=0.706, $p<10^{-454}$). D. M PSS regressor results: RP1 (r=0.70, $p<10^{-454}$), RP1 and RP3 ((r=0.701, $p<10^{-454}$)), RP1, RP3 and mRNA (r=0.702, $p<10^{-454}$). E. G1 PSS regressor results: RP1 (r=0.703, $p<10^{-454}$), RP1 and RP4 ((r=0.707, $p<10^{-454}$)), RP1, RP4 and mRNA (r=0.71, $p<10^{-454}$). F. M PSS regressor results: RP1 (r=0.701, $p<10^{-454}$), RP1 and RP4 ((r=0.71, $p<10^{-454}$)), RP1, RP4 and mRNA (r=0.712, $p<10^{-454}$). G. G1 PSS regressor results: RP2

(r=0.702, p<10^{-454}), RP2 and RP3 ((r=0.703, p<10^{-454})), RP2, RP3 and mRNA (r=0.706, p<10^{-454}). H. M PSS regressor results: RP2 (r=0.70, p<10^{-454}), RP2 and RP3 ((r=0.70, p<10^{-454})), RP2, RP3 and mRNA (r=0.701, p<10^{-454}). I. G1 PSS regressor results: RP2 (r=0.705, p<10^{-454}), RP2 and RP4 ((r=0.706, p<10^{-454})), RP2, RP4 and mRNA (r=0.71, p<10^{-454}). J. M PSS regressor results: RP2 (r=0.70, p<10^{-454}), RP2 and RP4 ((r=0.71, p<10^{-454})), RP2, RP4 and mRNA (r=0.712, p<10^{-454}). K. G1 PSS regressor results: RP3 (r=0.70, p<10^{-454}), RP3 and RP4 ((r=0.708, p<10^{-454})), RP3, RP4 and mRNA (r=0.713, p<10^{-454}). J. M PSS regressor results: RP3 (r=0.70, p<10^{-454}), RP3 and RP4 ((r=0.71, p<10^{-454})), RP3, RP4 and mRNA (r=0.715, p<10^{-454}).

In order to further demonstrate that each of the techniques, RP and PP, uncovers biologically relevant protein-protein interactions that cannot be detected by the other technique, three PPI network colouring schemes were defined, where "black" nodes represent differentially expressed (DE) genes between G1 and M phase of the cell cycle. In the first case, the black nodes were defined as genes that are DE according to RP but not based on PP (RP-PP); in the second case the black nodes were defined as genes that are DE according to PP but not based on RP (PP-RP); in the third case the black nodes were defined as genes that are DE according to both RP and PP; similarly to the previous analysis. We computed the mean distance (md) between all black nodes in each of the aforementioned three cases. Shorter distances between DE PPI nodes means more meaningful biological signals, as if indeed we uncover real regulatory changes in signalling pathways, we expect them to be clustered/close in the PPI network (we expect to see physical interactions between DE genes). The mean distance in the case of RP∩PP (125 genes) was shorter (2.01) than in the case of the RP-PP (999 genes) and the PP-RP (203 genes) groups (2.12 and 2.13, respectively), depicted in Figure 7 of the main text. We re-executed this analysis, only now instead of calculating the RP DE genes according to all four replicates, we calculated them according to all pairs of replicates, resulting in 6 DE groups, and then utilized the 3 non-overlapping ones instead of the PP and averaged RP, resulting in 3 independent analyses (expressly employing DE genes based on [Rep1 and Rep2] and [Rep3 and Rep4], [Rep1 and Rep3] and [Rep2 and Rep4], [Rep1 and Rep4] and [Rep2 and Rep3]). The md results are: for [Rep1 and Rep2] and [Rep3 and Rep4] DE based groups, which we will name RP1 and RP2 respectively: RP1∩RP2: 2.11 (171 genes), RP1-RP2: 2.47 (978 genes), RP2-RP1: 2.15 (864 genes); [Rep1 and Rep3] and [Rep2 and Rep4] DE based groups, which we will name RP3 and RP4 respectively:

RP3∩RP4: 2.17 (159 genes), RP3-RP4: 2.14 (970 genes), RP4-RP3: 2.38 (882 genes); and [Rep1 and Rep4] and [Rep2 and Rep3] DE based groups, which we will name RP5 and RP6 respectively:

RP5∩RP6: 2.17 (138 genes), RP5-RP6: 2.42 (900 genes), RP6-RP5: 2.10 (1000 genes). As can be seen, in most of the cases the intersection does not achieve the shortest distance (as in the case of RP vs. PP), supporting the conjecture that the relations reported in main text are not trivially due variation among replicas. To empirically test the significance of the shorter distance achieved by combing PP and RP, as opposed to using only technical replicates of RP, we devised the following empirical p-value: since RP1∩RP2 attained the shortest distance (which is 2.11), we sampled uniformly at random 125 genes from the PPI network 1000 times, and computed the mean distance between them, mdi, the p-value being (# of times (2.11 - mdi) ≤ (2.11 - 2.01))/1000, which is indeed < 0.001.

At the next step our objective was to show that both PP and RP can be used for detecting relevant differentially transcriptional and post transcriptional regulated genes, and that each of these methods exclusively detects relevant genes. We performed pathway and biological process enrichment for each of the DE groups, 1. RP∩PP (125 genes). 2. RP-PP (1,090 genes). 3. PP-RP (200 genes). To achieve our objective, we aimed to show that relevant pathways and biological processes are significantly enriched with DE genes in all three cases, see Supplementary

Information Table 1 (section 2.2) above (Figure 6 of the main text includes selected pathways and biological processes (DAVID analysis) which are significantly enriched by the 3 groups of DE genes, here we examine only our pathway enrichment analysis). Using the same DE groups as in the above PPI analysis, we compared the pathway enrichment results of RP∩PP, with that of RP1∩RP2, RP3∩RP4, RP5∩RP6, taking only pathways enriched by at least two of the groups and that passed FDR. The results are summarized in table 3 below, the p-values reported for the RP groups are based on the average, as can clearly be seen, utilizing RP∩PP uncovers more significant and relevant pathways.

Table 3: Comparison of pathway enrichment utilizing both RP and PP, as opposed to only RP replicates.

RP∩PP	p	RPi∩RPj	p
Cell cycle	1.9e-13	Cell cycle	0.0014
DNA Replication	8.3e-06	Electron Transport Chain	2.3e-06
Cytoplasmic Ribosomal Proteins	3.8e-05	Epithelium TarBase	1.7e-05
G1 to S cell cycle control	1.1e-04	Hypertrophy Model	0.0009
		MAPK signaling pathway	10e-05

3.6 RP, PP, and mRNA Pearson correlation with PSS

In order to be comparable to previous studies which tried to estimate how much of the variance of steady state protein levels (PSS) can be explained by mRNA levels, and which performed Pearson correlations [11-13] (as opposed to the Spearman correlations performed throughout our study), we calculated the Pearson correlations between steady state protein levels and RP, PP and mRNA levels respectively. In our opinion it is more ‘correct’ to employ Spearman correlations which unlike Pearson do not assume linearity, as when comparing mRNA levels with ribosomal density and protein levels that means that we assume there is no translation regulation, which is known to be incorrect. Moreover, even if the relationship was linear, since all experimental measurements have a saturation range, that linear relationship would have been distorted.

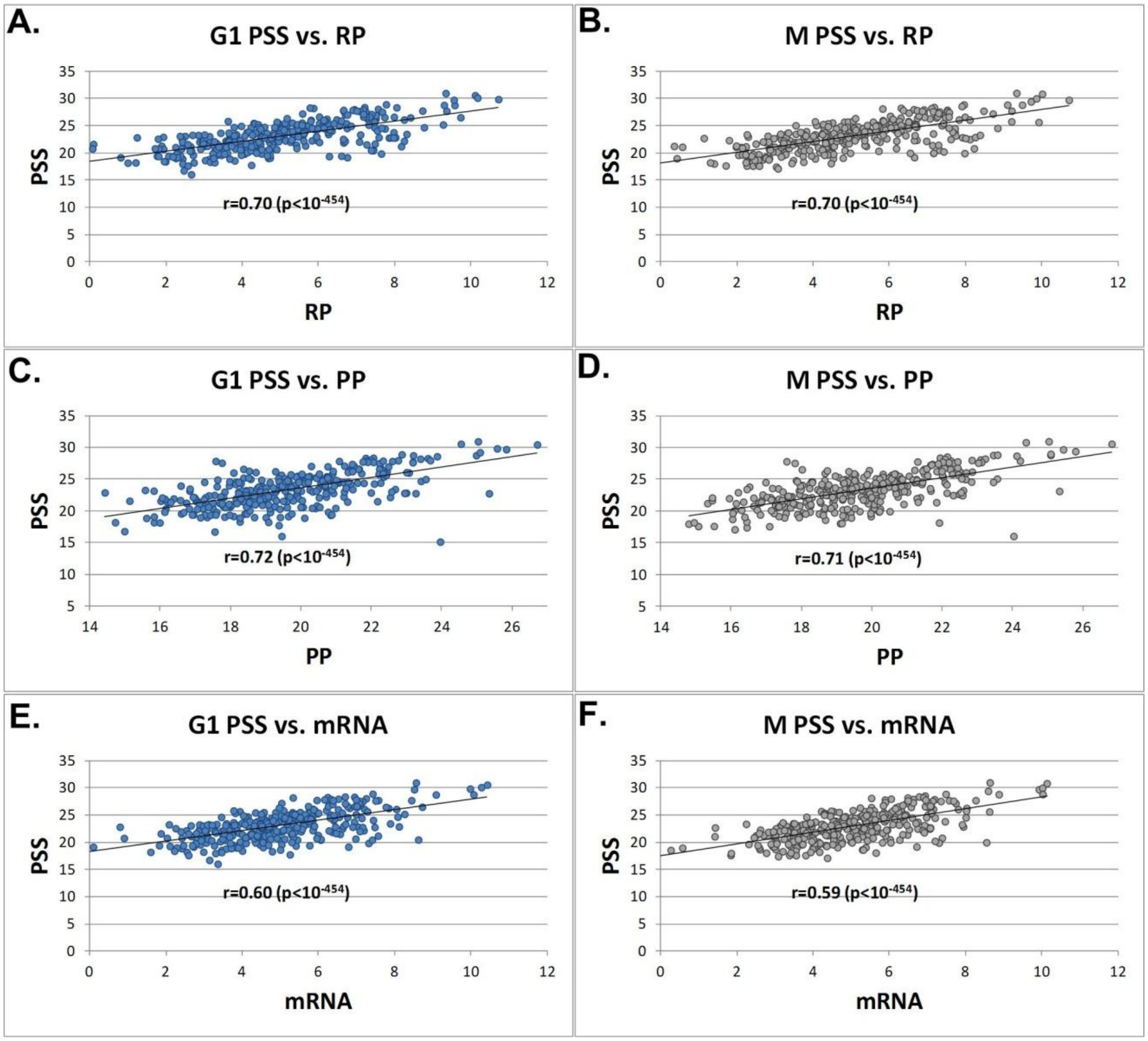

Figure 11: Pearson correlations for: A. Dot plot of steady state protein levels (PSS) (y-axis log2(intensity), data is log2-scaled) and Ribo-Seq (RP) (x-axis, read count log2-scaled RPKM (see main text Methods)) G1 phase. B. Dot plot of PSS (y-axis [need to add units], data is log2-scaled) and RP levels (x-axis, read count log2-scaled RPKM (see main text Methods)) M phase. C. Dot plot of steady state protein levels (PSS) (y-axis log2(intensity), data is log2-scaled) and PUNCH-P (PP) (x-axis [need to add units], data is log2-scaled) G1 phase. D. Dot plot of PSS (y-axis [need to add units], data is log2-scaled) and PP levels (y-axis [need to add units], data is log2-scaled) M phase. E. Dot plot of steady state protein levels (PSS) (y-axis log2(intensity), data is log2-scaled) and mRNA levels (x-axis, log2-scaled RPKM (see main text Methods)) G1 phase. F. Dot plot of PSS (y-axis log2(intensity), data is log2-scaled) and mRNA levels (x-axis, log2-scaled RPKM (see main text Methods)) M phase.

3.7 Supplementary Tables Description

Regressor correlations of PP, RP, and mRNA with PSS can be found in Supplementary_Table_S1_RegressorCorrs.xlsx.
Signalling pathways can be found in supplementary file Supplementary_Table_S2_Human_Pathways.xlsx.
Biological process enrichment for: 1. RP-PP (genes that are significantly DE in RP but not in PP) can be found in Supplementary_Table_S3_RPDavidReports.xlsx. 2. PP-RP (genes that are significantly DE in PP but not in RP) can be found in Supplementary_Table_S4_PPDavidReports.xlsx. 3. RP∩PP (genes that are significantly DE both in PP and in RP) can be found in Supplementary_Table_S5_RPiPPDavidReports.xlsx.
Protein-Protein Interactions clustering analysis can be found in Supplementary_Table_S6_ClusterPathwayEnrichment.xlsx.
Ribo-Seq and PUNCH-P differentially expressed genes in opposite directions can be found in Supplementary_Table_S7_RPopPPdiffGenes.xlsx.
Ribo-Seq and PUNCH-P data can be found in Supplementary_Table_S8_RP_PP_Data.xlsx:
Sheet RP Reps: Contains the mean footprint read count per replicate per gene, and since reads were mapped to transcripts, the read count was calculated as the sum of the reads mapped to each transcript as described above. In all our analyses we included only genes with read count > 0, but here for the readers convenience we supply the read counts for all the genes.
Sheet mRNA Reps: Contains the mean read count per replicate per gene.
Sheet Read Stats Legend: a legend defining the read groups for the 2 sheets below describing RP and mRNA read statistics.
Sheet RP Read stats: the total number of reads, number of reads mapped to rRNA, number of reads mapped to tRNA, total number of viable reads, number of reads mapped, and number of multi reads, per RP replicate.
Sheet mRNA Read stats: the total number of reads, number of reads mapped to rRNA, number of reads mapped to tRNA, total number of viable reads, number of reads mapped, and number of multi reads, per mRNA replicate.
Sheet PP Reps: Contains both the iBAQ and LFQ normalized PUNCH-P data per replicate for the readers convenience.
Sheet PSS Reps: Contains both the iBAQ and LFQ normalized steady state protein levels data per replicate for the readers convenience.
Sheet RP Fold-Change: Contains the M/G1 RP fold change, p-values, and FDR p-values (calculated according to [72]), as calculated according to [36] (described above and in the supplementary methods), sorted according to FDR p-values. We reiterate that only genes with read count > 0 were included in the analysis (resulting in 12514 genes), and the RP DE genes were selected according to the lowest 10% FDR p-values.
Sheet PP Fold-Change: Contains M/G1 PP fold change and ANOVA p-values, sorted according to the p-values (there are 3620 genes with measurements in both M and G1). We reiterate (as described above) that PP differentially expressed genes between M and G1 are determined according to highest significant (ANOVA) fold-change. The top 10% highest significant fold change was selected as PP DE.
Supplementary_Table_S9_RPiPP_ClusterPEDetails.xlsx contains details of the module inference/clustering analysis performed based on protein-protein interactions among genes detected to be differentially expressed both based on PP and based on RP.

4 References

1. Newman, M.E., *Modularity and community structure in networks.* Proceedings of the National Academy of Sciences, 2006. **103**(23): p. 8577-8582.
2. Anders, S. and W. Huber, *Differential expression analysis for sequence count data.* Genome Biol, 2010. **11**(10): p. R106.
3. Marioni, J.C., et al., *RNA-seq: an assessment of technical reproducibility and comparison with gene expression arrays.* Genome Research, 2008. **18**(9): p. 1509-1517.
4. Wang, L., et al., *DEGseq: an R package for identifying differentially expressed genes from RNA-seq data.* Bioinformatics, 2010. **26**(1): p. 136-138.
5. Nagalakshmi, U., et al., *The transcriptional landscape of the yeast genome defined by RNA sequencing.* Science, 2008. **320**(5881): p. 1344-9.
6. Robinson, M.D. and G.K. Smyth, *Moderated statistical tests for assessing differences in tag abundance.* Bioinformatics, 2007. **23**(21): p. 2881-2887.
7. Whitaker, L., *On the Poisson law of small numbers.* Biometrika, 1914. **10**(1): p. 36-71.
8. Robinson, M.D., D.J. McCarthy, and G.K. Smyth, *edgeR: a Bioconductor package for differential expression analysis of digital gene expression data.* Bioinformatics, 2010. **26**(1): p. 139-140.
9. Robinson, M.D. and G.K. Smyth, *Small-sample estimation of negative binomial dispersion, with applications to SAGE data.* Biostatistics, 2008. **9**(2): p. 321-332.
10. Benjamini, Y. and Y. Hochberg, *Controlling the false discovery rate: a practical and powerful approach to multiple testing.* Journal of the Royal Statistical Society. Series B (Methodological), 1995: p. 289-300.
11. Schwanhäusser, B., et al., *Global quantification of mammalian gene expression control.* Nature, 2011. **473**(7347): p. 337-342.
12. Vogel, C., et al., *Sequence signatures and mRNA concentration can explain two-thirds of protein abundance variation in a human cell line.* Molecular Systems Biology, 2010. **6**(1).
13. Low, T.Y., et al., *Quantitative and qualitative proteome characteristics extracted from in-depth integrated genomics and proteomics analysis.* Cell reports, 2013. **5**(5): p. 1469-1478.
